# Association of Polymorphisms in Pharmacogenetic Candidate Genes (OPRD1, GAL, ABCB1, OPRM1) with Opioid Dependence in European Population: A Case-Control Study

**DOI:** 10.1371/journal.pone.0075359

**Published:** 2013-09-25

**Authors:** Beate Beer, Robert Erb, Marion Pavlic, Hanno Ulmer, Salvatore Giacomuzzi, Yvonne Riemer, Herbert Oberacher

**Affiliations:** 1 Institute of Legal Medicine, Innsbruck Medical University, Innsbruck, Austria; 2 Department of Medical Statistics, Informatics, and Health Economics, Innsbruck Medical University, Innsbruck, Austria; 3 Department of General Psychiatry, Innsbruck Medical University, Innsbruck, Austria; Peking University, China

## Abstract

It is becoming increasingly evident that genetic variants contribute to the development of opioid addiction. An elucidation of these genetic factors is crucial for a better understanding of this chronic disease and may help to develop novel therapeutic strategies. In recent years, several candidate genes were implicated in opioid dependence. However, most study findings have not been replicated and additional studies are required before reported associations can be considered robust. Thus, the major objective of this study was to replicate earlier findings and to identify new genetic polymorphisms contributing to the individual susceptibility to opioid addiction, respectively. Therefore, a candidate gene association study was conducted including 142 well-phenotyped long-term opioid addicts undergoing opioid maintenance therapy and 142 well-matched healthy controls. In both study groups, 24 single nucleotide polymorphisms predominantly located in pharmacogenetic candidate genes have been genotyped using an accurate mass spectrometry based method. The most significant associations with opioid addiction (remaining significant after adjustment for multiple testing) were observed for the rs948854 SNP in the galanin gene (*GAL*, p = 0.001) and the rs2236861 SNP in the delta opioid receptor gene (*OPRD1*, p = 0.001). Moreover, an association of the ATP binding cassette transporter 1 (*ABCB1*) variant rs1045642 and the Mu Opioid receptor (*OPRM1*) variant rs9479757 with opioid addiction was observed. The present study provides further support for a contribution of *GAL* and *OPRD1* variants to the development of opioid addiction. Furthermore, our results indicate a potential contribution of *OPRM1* and *ABCB1* SNPs to the development of this chronic relapsing disease. Therefore it seems important that these genes are addressed in further addiction related studies.

## Introduction

The etiology of opioid addiction is complex and involves environmental, psychological as well as drug induced factors [1,2]. Moreover, genetic factors play an essential role in the pathogenesis of opioid addiction - estimates of the heritability of opioid abuse and/or addiction range from 43 to 60% [3,4]. Several candidate genes have been implicated in opioid dependence. One of the most extensively studied candidates is the mu opioid receptor gene (*OPRM1*) and its functional single nucleotide polymorphism (SNP) rs1799971 (A118G), which has been reported to affect the individual vulnerability to opioid dependence, although not in all studies [5]. Furthermore, also other genes of the opioid system or genes involved in serotoninergic, dopaminergic or noradrenergic pathways have been linked with this chronic relapsing disease [1,2,5-8]. However, study findings have often not been replicated or are inconsistent between studies and the overall knowledge on genetic factors influencing the development of opioid addiction is still limited [3,5,6]. Moreover, many study results are based on the analysis of populations with mixed ancestries and/or poorly phenotyped cohorts [6,7]. Thus, further research is required to identify genetic variants contributing to the development of opioid addiction, to confirm tentative genetic associations and to improve the neurobiological understanding of opioid dependence [1,6].

The major aim of the present study was to identify genetic polymorphisms contributing to the individual susceptibility to opioid addiction and to replicate earlier findings in this regard, respectively. Therefore, a candidate gene case-control association study including 14 different genes and 24 SNPs was performed. A particular focus was put on pharmacogenetic candidate genes, which may contribute to inter-individual variations in opioid effects and opioid-induced behaviours (i.e. genes encoding proteins directly involved in the pharmacokinetic and pharmacodynamic action of commonly abused opioids) [1,6,9]. A well-phenotyped sample of long-term addicts undergoing opioid maintenance treatment (OMT) and a well-matched sample of healthy controls, both recruited according to stringent exclusion criteria, were investigated. Genotyping was performed using a multiplex polymerase chain reaction (PCR) assay combined with ion-pair reversed-phase high-performance liquid chromatography-electrospray ionization mass spectrometry (ICEMS). Thereby, significant associations of pharmacogenetic variants with opioid addiction were identified and preliminary, yet unconfirmed genetic associations were replicated.

## Patients and Methods

### Ethics statement

The present study was approved by the local Ethics Committee of the Innsbruck Medical University and carried out in accordance with the Declaration of Helsinki as adopted and promulgated by the National Institutes of Health and the European Union. All participants signed an informed consent after receiving a complete description of the study and been given the chance to discuss any questions or issues.

### Study design and study subjects

The presented study is a prospective observational cohort study that was originally intended to be a sub-study of a clinical trial. The clinical trial, EudraCT registration number 2008-002714-22, did not proceed. Participants described here did not take part in a clinical trial, and were recruited based on the inclusion criteria of substudy 1 of our study protocol (Protocol S1). For the presented candidate gene association study, a total of 148 unrelated opioid dependent individuals undergoing opioid maintenance treatment were recruited at the drug addiction outpatient clinic of the Innsbruck Medical University. Following inclusion criteria were applied: (1) written informed consent; (2) opioid dependence according to the DSM-IV criteria; (3) opioid maintenance therapy with either methadone or buprenorphine; (4) history of at least 2 years of daily heroin (and/or morphine) use. Patients were not included if they met one of the following exclusion criteria: (1) alcohol dependence; (2) regular co-use of cocaine; (3) age < 18 and > 50 years; (4) opioid dependence < 2 years. Ascertainment was made by a structured personal interview, using a self-designed questionnaire for data collection. All interviews were conducted by the same physician. The 142 healthy control subjects were included after personal interview and according to following exclusion criteria: (1) age < 18 and > 50 years; (2) history of alcohol dependence (3) history of regular illicit drug use. The controls were matched for ethnicity, sex and age to the patient sample.

### Genotyping

#### DNA sample collection and DNA extraction

DNA samples of all participants (all of Western European descent, 96% Austrian) were obtained via buccal swabs. DNA was extracted using a standard Chelex extraction protocol [10]. The DNA content of the samples was determined by spectrophotometry (ND-1000, Nano-Drop, Wilmington, USA) and values between 15-121 ng/µl were determined.

#### Selection of polymorphic loci

The present study involved a candidate gene approach. The selection of the genes and polymorphic loci was performed after an extensive literature study and was primarily based on the knowledge of metabolic pathways, transporters or targets of opioids. The main criterion for the inclusion of a genetic variant was the availability of data suggesting or confirming a functional relevance. In total, 24 single nucleotide polymorphisms (SNPs) located in 14 different genes were selected (Table 1). Four of the 14 investigated genes are postulated to be involved in opioid pharmacokinetics (*ABCB1*, *SLCO1B1*, *SLCO1A2*, *UGT2B7*) [11,12], eight genes are postulated to be directly or indirectly involved in the pharmacodynamic opioid response (*OPRM1*, *OPRD1*, *OPRK1*, *GAL*, *STAT6*, *ADRBK2*, *COMT*, *MC1R*) [7,8,12-14] and two genes were reported to be associated with opioid addiction or opioid treatment outcome in previous studies (*DRD2*, *HTR1A*), although not directly involved in opioid pharmacodynamics and kinetics [15,16].

**Table 1:** List of the selected candidate genes and polymorphisms.

**Symbol**	**Gene**	**SNP ID**	**Alleles**	**Frequency^1^**
*OPRM1*	Opioid receptor, mu 1	rs1799971	A/G	0.14
		rs9479757	G/A	0.08
		rs3778151	T/C	0.15
		rs510769	G/A	0.25
*OPRD1*	Opioid receptor, delta 1	rs2236861	C/T	0.29
		rs1042114	T/G	0.11
*OPRK1*	Opioid receptor, kappa 1	rs1051660	G/T	0.08
*ADRBK2*	Beta-adrenergic receptor kinase 2	rs5761122	G/A	0.29
*STAT6*	Signal transducer and activator of transcription 6	rs841718	T/C	0.47
*COMT*	Catechol-O-methyltransferase	rs4680	G/A	0.49
*MC1R*	Melanocortin 1 receptor	rs1805007	C/T	0.05
		rs1805008	C/T	0.07
*UGT2B7*	Uridine diphosphate glycosyltransferase 2B7	rs7439366	T/C	0.43
*ABCB1*	ATP-binding cassette, subfamily B, member 1	rs2032588	C/T	0.07
		rs2032582	G/T/A	0.40 (T)
				0.02 (A)
		rs1045642	C/T	0.48
*SLCO1B1*	Solute carrier organic anion transporter family, 1B1	rs4149056	T/C	0.15
		rs2306283	T/C	0.35
*SCLO1A2*	Solute carrier organic anion transporter family, 1A2	rs11568563	A/C	0.07
		rs45502302	A/T	0
*GAL*	Galanin	rs948854	A/G	0.23
*HTR1A*	5-Hydroxytryptamine receptor 1A	rs6295	C/G	0.49
*DRD2*	Dopamine receptor D2	rs6275	C/T	0.32
		rs6277	T/C	0.43

1 Minor allele frequency observed in the control group (n = 142).

#### PCR-ICEMS genotyping

Genotyping of the 24 polymorphic loci was performed using a multiplex PCR assay combined with ion-pair reversed-phase high-performance liquid chromatography-electrospray ionization mass spectrometry (ICEMS) [17,18]. Details of the genotyping assay are described elsewhere (Supporting Information S1) [19]. In brief, a 2-step PCR approach covering 23 polymorphic sites was developed using hybrid primers consisting of a target-specific and a non-specific universal sequence. The thereby generated PCR products were directly analyzed by ICEMS. One SNP (*DRD2* rs6277) was analyzed in singleplex reactions, since its close genomic vicinity to the *DRD2* rs6275 SNP did not allow for a successful integration into the multiplex assay. A total of 399 SNPs were retyped by Sanger sequencing and a concordance rate of 99.8% was assessed.

### Statistical analysis

The sample size of the study was calculated aiming to detect odds ratios of approximately 2 and higher with 80% statistical power and a significance level of p < 0.05. Because of varying prevalences of the genotypes/alleles a definite single sample size estimation was not possible. An odds ratio of 2 means for instance that the control proportion is 0.52 and the case proportion is 0.69 or that the control proportion is 0.25 and the case proportion is 0.4. All statistical analyses were performed with the PASW Statistics 18 software package. Differences in sociodemographic and clinical characteristics between the groups were assessed by the Pearson’s chi-square and Fisher’s exact tests. Furthermore, Chi-square and Fisher’s exact testing were applied to assess deviations from Hardy-Weinberg equilibrium, to compare allele and genotype frequencies between cases and controls and to estimate odds ratios with their corresponding 95% confidence intervals. For certain statistical purposes, the *ABCB1* SNPs rs2032582 and rs1045642 were tabulated as 2-locus genotype [20] and those genotype patterns with a low frequency (< 0.05) were pooled into a single class called “others”. Beside overall p-values (df = 2), point-wise p-values (df = 1) were calculated by comparing the genotype frequency of each genotype subgroup to the pooled genotype frequency of the remaining subgroups. Finally, the results were adjusted for multiple testing by using the conservative Bonferroni correction (p-value/ number of tested SNPs, significance level p < 0.002).

## Results

### Sociodemographic and clinical characteristics of the study subjects

In total, 142 of the 148 recruited patients and all 142 healthy control subjects were included in the study. One patient was excluded due to regular co-use of cocaine. Five patients were excluded due to other reasons (discontinuation of treatment, no saliva swab provided, lack of phenotypic data). All study participants were of Western European descent and 96% were Austrian. There were no significant differences between patients and controls in terms of age, sex or ethnicity. The patient group consisted of 80 individuals undergoing methadone maintenance treatment (MMT) and 62 individuals undergoing buprenorphine maintenance treatment (BMT). The mean duration of opioid dependence was 11 years (long-term addicts). Complete sociodemographic and clinical data were recorded for 89% of all patients. For the remaining patients the major part of the data was available. The key sociodemographic and clinical characteristics are summarized in Table 2. The daily maintenance doses of methadone ranged between 10 mg and 150 mg, the daily maintenance doses of buprenorphine ranged between 0.4 mg and 32 mg. In the MMT group significantly more non-abstinent patients with regard to illicit opioid use were identified than in the BMT group (p = 0.017, χ^2^ = 5.75, df = 1). The observed prevalence of urines positive for illicit opioids was comparable to the data reported in other studies [21].

**Table 2:** Main sociodemographic data of all study subjects (n = 284) and key clinical characteristics of the patients undergoing methadone or buprenorphine maintenance treatment.

	**Patients MMT**	**Patients BMT**	**Patients total**	**Controls**
Number	80	62	142	142
Age in years, mean (SD±)	33 (8)	29 (8)	31 (8)	29 (6)
Male %	70	65	68	57
Western European descent %	100	100	100	100
Body mass index, mean (SD±)	23.5 (4)	22.1 (4)	22.9 (4)	
Mean dose, M or B in mg (SD±)	77 (39)	14 (9)		
Opioid abuse in years, mean (SD±)	13 (8)	8 (6)	11 (8)	
Duration of OMT in month, mean (SD±)	85 (70)	54 (48)	72 (64)	
Illicit opioid use				
Abstinence^1^ (%)	28 (35)	32 (52)	60 (42)	
Non-abstinence^2^ (%)	51 (64)	25 (40)	76 (54)	
No data available (%)	1 (1)	5 (8)	6 (4)	

^1^ ≤ 2/10 opioid positive urines

^2^ >2/10 opioid positive urines.

M, methadone; B, buprenorphine; OMT, opioid maintenance treatment; SD, standard deviation.

### Genotyping results

In the present study, 6816 SNPs (24 SNPs in 14 candidate genes, 284 individuals) were genotyped using PCR-ICEMS. For all study subjects, complete and high quality genotype data were acquired. Confirmative sequencing experiments revealed that the applied genotyping approach is accurate. The assessed genotype and allele frequencies (Table 1) correlated well with published frequencies for other European populations (http://www.ncbi.nlm.nih.gov/snp/). In the control group, the genotype distribution of one polymorphism (*ADRBK2* rs5761122) showed a statistically significant deviation from the Hardy-Weinberg equilibrium (p = 0.001, χ^2^ = 10.88). In the patient group, chi-square testing revealed a statistically significant deviation of the genotype distribution of two polymorphisms: *GAL* rs948854 (p = 0.0002, χ^2^ = 14.34) and *ABCB1* rs1045642 (p = 0.002, χ^2^ = 9.35). One investigated SNP (*SCLO1A2*, rs45502302) turned out to be not polymorphic in our samples and was therefore excluded from the statistical analysis.

When the genotype and allele frequencies of the patient group were compared to those of the control group, significant differences could be observed for SNPs in the genes *OPRD1*, *GAL*, *ABCB1* and *OPRM1*. The observed (overall and point-wise) p-values, odds ratios and 95% confidence intervals are summarized in Tables 3 and 4. No significant sex-specific differences were found.

**Table 3:** Association between genotypes/alleles and opioid dependence (patients: n = 142, controls: n = 142).

**Genotypes/Alleles**	**Patients**, n (frequency)	**Controls**, n (frequency)	**OR (95% CI**)	**Point-wise p** ^1^	**Overall p** ^2^
*OPRD1* rs2236861					
CC	97 (0.68)	74 (0.52)	1.98 (1.19-3.31)	0.005	0.004
CT	42 (0.30)	55 (0.39)	0.66 (0.39-1.12)	0.104	
TT	3 (0.02)	13 (0.09)	0.21 (0.05-0.83)	0.018	
C	236 (0.83)	203 (0.72)			0.001
T	48 (0.17)	81 (0.29)			
*GAL* rs948854					
AA	87 (0.61)	81 (0.57)	1.19 (0.72-1.97)	0.469	0.001
AG	37 (0.26)	57 (0.40)	0.53 (0.31-0.90)	0.012	
GG	18 (0.13)	4 (0.03)	5.01 (1.54-18.00)	0.002	
A	211 (0.74)	219 (0.77)			0.434
G	73 (0.26)	65 (0.23)			
*ABCB1* rs1045642					
CC	23 (0.16)	40 (0.28)	0.49 (0.27-0.91)	0.015	0.026
CT	89 (0.63)	69 (0.49)	1.78 (1.08-2.93)	0.017	
TT	30 (0.21)	33 (0.23)	0.89 (0.49-1.61)	0.668	
C	135 (0.48)	149 (0.53)			0.240
T	149 (0.53)	135 (0.48)			
*OPRM1* rs9479757					
GG	130 (0.92)	119 (0.84)	2.09 (0.95-4.69)	0.047	0.036
GA	11 (0.8)	23 (0.16)	0.43 (0.19-0.98)	0.028	
AA	1 (0.01)	0 (0)		1.000	
G	171 (0.95)	261 (0.92)			0.085
A	13 (0.05)	23 (0.08)			

^1^ χ^2^ analysis or fisher’s exact test, df =1; ^2^ χ^2^ analysis or fisher’s exact test, df =2; OR, Odds ratio; CI, confidence interval.

**Table 4:** Association of the *ABCB1* rs2032582 - rs1045642 genotype patterns with opioid dependence (patients: n = 142, controls: n = 142).

**2-Locus Genotype**	**Patients**, n (frequency)	**Controls**, n (frequency)	**OR (95% CI**)	**Point-wise p** ^1^	**Overall p** ^2^
GG-CC	18 (0.13)	33 (0.23)	0.48 (0.24-0.94)	0.020	
GG-CT	24 (0.17)	15 (0.11)	1.72 (0.82-3.64)	0.121	
GT-CT	62 (0.44)	47 (0.33)	1.57 (0.94-2.61)	0.067	
TT-TT	18 (0.13)	19 (0.13)	0.94 (0.45-1.98)	0.860	
Others	20 (0.14)	28 (0.20)	0.67 (0.34-1.31)	0.205	0.042

^1^ χ^2^ analysis or fisher’s exact test, df =1; ^2^ χ^2^ analysis or fisher’s exact test, df =2; OR, Odds ratio; CI, confidence interval.

#### OPRD1, rs223686

We observed significantly more homozygous carriers of the *OPRD1* rs2236861 major allele (CC genotype) in the patient group and more homozygous carriers of the minor allele (TT genotypes) in the control group (p = 0.004, χ^2^ = 11.09, df = 2). Correspondingly, we found a significantly higher T allele frequency in the controls (p = 0.001, χ^2^ = 10.92, df = 1). The latter finding withstood the Bonferroni correction for multiple testing.

#### GAL, rs948854

For this *GAL* SNP significant genotype frequency differences where observed, which also remained significant after adjustment for multiple testing (p = 0.001, χ^2^ = 13.38, df = 2). Homozygous carriers of the minor allele (GG genotype) were significantly more prevalent in the patient group (p = 0.002, χ^2^ = 9.657, df = 1). At the same time, less AG carriers where detected in the patient group. The overall allele frequencies showed no significant differences between the two groups.

#### ABCB1, rs1045642, rs2032582

We found a higher prevalence of CT genotypes and less CC carriers of the *ABCB1* rs1045642 variant in the patient group (p = 0.026, χ^2^ = 7.26, df = 2). Two SNPs in the *ABCB1* (rs2032582 and rs1045642) were additionally evaluated as 2-locus genotype pattern (Table 4), since they have been found to be in strong linkage disequilibrium [19]. Among the opioid dependent study participants, a significantly lower frequency of GG-CC genotype patterns was observed (p = 0.020, χ^2^ = 5.377, df = 1).

#### OPRM1, rs9479757

Regarding the *OPRM1* rs9479757 SNP we found a significantly lower GA genotype frequency (p = 0.028, χ^2^ = 4.81, df = 1) and a higher GG genotype frequency in the patients compared to the controls.

## Discussion

In the present study, a well-phenotyped sample of long-term opioid addicts and a sample of well-matched controls (all of Western European descent) were investigated. By using a candidate gene approach, significant associations of pharmacogenetic variants with opioid addiction were identified and preliminary, yet unconfirmed genetic associations were replicated.

When comparing genotype and allele frequencies of the patient group with those of the control group the lowest overall p-values (remaining significant after Bonferroni correction) were observed for SNPs located in the *OPRD1* (rs2236861) and *GAL* (rs948854). Significant frequency differences were also observed for an *ABCB1* SNP (rs1045642), a *ABCB1* 2-locus genotype pattern (rs2032582 - rs1045642), and a SNP located in the *OPRM1* gene (rs9479757).

### Delta opioid receptor (OPRD1, chromosome 1)

Opioid receptors can be subdivided into mu-, kappa- and delta opioid receptors. The mu opioid receptor is the primary target of commonly abused opioids and has attracted major attention in addiction related studies [5,6]. However, also the delta opioid receptor is a plausible candidate for case-control studies, since it has been shown to be involved in rewarding and analgesic effects of opioids and the development of opioid tolerance [22,23]. Indeed, we observed a significant association of the intronic *OPRD1* SNP rs2236861 with opioid dependence in our European study population. This finding supports the results of a candidate gene association study that also found the rs2236861 SNP to be positively associated with opioid addiction in European Americans [7]. Also a very recent large case-control study found a positive association of this SNP with heroin dependence in an Australian study population (primarily of European ancestry) [23]. These facts strongly indicate an important role of this SNP in addiction susceptibility. However, the functional consequence of this SNP still remains to be elucidated. The non-synonymous *OPRD1* SNP rs1042114, which was associated with opioid dependence risk in an European American study population [24], was not found to be positively associated with opioid dependence in our study (nor in the study of Nelson and co-workers [23]).

### *Galanin* (*GAL, chromosome 11*).

Galanin is a 30-amino acid neuropeptide which is widely distributed in the central as well as peripheral nervous system. It has been indicated to be involved in diverse behavioural functions and stress response. Furthermore, *GAL* has been linked with neurochemical and behavioural effects of opiates and opioid withdrawal [25,26]. However, its role as susceptibility factor in opioid addiction has hardly been studied [7]. The SNP (rs948854) we investigated in our approach is located in the *GAL* promoter region and has been repeatedly linked with symptom severity and hypothalamic-pituitary-adrenal-axis activity in female patients suffering from panic and other anxiety disorders [27,28]. More precisely, the minor allele (G) was associated with more severe anxiety pathology and a higher activity of the hypothalamic-pituitary-adrenal-axis, thereby supporting a functional role of this SNP in the pathophysiology of psychiatric phenotypes. By investigating this SNP in context with opioid addiction, we found significantly more homozygous carriers of the minor allele (G) among the patients. This association remained significant after adjustment for multiple testing. To our knowledge, a genetic association of this promoter SNP with opioid addiction has not been described so far. The presented result may significantly contribute to current knowledge of the role of GAL in opioid addiction susceptibility, which so far was mainly based on one study that found a *GAL* variant located in intron 2 (rs694066) to be associated with heroin addiction [7].

### P-glycoprotein (ABCB1, chromosome 7)

The p-glycoprotein belongs to the ATP binding cassette transporter family and acts as a multispecific efflux pump transporting various endogenous compounds as well as drugs from the intracellular to the extracellular domain [29]. P-glycoprotein is expressed in intestinal, kidney or hepatic cells as well as endothelial cells of brain capillaries (blood brain barrier) and is suggested to play a critical role in the distribution of drugs including certain opioids [12,30]. A functional impairment of the p-glycoprotein mediated drug transport is expected to result in an increased oral bioavailability, reduced renal clearance and increased brain concentrations of its substrates [31]. Different SNPs as well as haplo- and genotype patterns of the *ABCB1* have been linked with the level of expression and/or function of the p-glycoprotein [31,32]. Many studies focused on the SNP rs1045642 (exon 26), which is a common variant in the coding region of the *ABCB1* gene. The T variant of this SNP has been associated with impaired function and/or expression of the p-glycoprotein in vitro and in vivo [33]. In a recent study investigating 98 methadone maintained patients, an *ABCB1* multilocus genotype pattern including this SNP (rs1045642- rs2032582-rs1128503 TT-TT-TT, and by trend also TT-GT-CT) was linked to the requirements of “higher” methadone doses (> 150 mg/day) for treatment stabilization [34]. Two further studies point to a significant role of these *ABCB1* SNPs for opioid dosage requirements [15,35] while other studies did not find any relationship [36,37]. We found in our study a significantly higher frequency of the rs1045642 CT genotype and a lower frequency of CC genotypes in the patient group compared to the controls. Moreover, a trend towards a higher frequency of the 2-locus genotype pattern (rs2032582 - rs1045642) GT-CT and significantly less GG-CC genotypes (linked to a “normal” p-glycoprotein activity) were observed among the addicts. The role of the p-glycoprotein as susceptibility factor has already been discussed in context with other psychiatric disorders and it has been hypothesized, that an impaired functionality of the p-glycoprotein may lead to an increased accumulation of particular endogenous compounds (e.g. cortisol) in the brain, which are related to psychiatric disorders such as depression [38,39]. Principally, a similar mechanism could also underlie opioid addiction susceptibility.

### Mu opioid receptor (OPRM1, chromosome 6)

Many endogenous and exogenous and opioids exert their primary effects at the mu opioid receptor. Correspondingly, numerous studies focussed on the association of *OPRM1* polymorphisms with opioid addiction [2,7]. The most extensively studied *OPRM1* variant in this context is the non-synonymous and functional SNP rs1799971 (A118G). However, the studies on this variant brought controversial results [6,7,40,41]. In our European study population, no significant association of this SNP with opioid addiction was found (p = 0.27). However, we found significant frequency differences of the *OPRM1* rs9479757 (IVS2 + 31G/A, intron 2) genotypes between cases and controls - the GG genotype was more prevalent among the cases. This SNP is a rarely investigated variant and data on its functional consequence are practically lacking [42]. There is one study that found an overrepresentation of the G variant (as part of a haplotype) in regular smokers compared to non-smokers [43], which also points to a potential contribution of this SNP to addictive behaviour. However, further research is required to verify the role of this SNP in addiction susceptibility.

A limitation of the present study is the relatively small sample size. However, the most significant genetic associations we observed (*OPRD1*, *GAL*) were in accordance with previous reports based on larger sample sizes [7,23]. A major strength of this study is that only well-characterized individuals, who were prospectively recruited according to stringent exclusion criteria, were included. A total of 96% of all individuals were of Austrian ancestry and patients and controls were well-matched in terms of age, sex and ancestry. Moreover, an accurate genotyping method has been applied in the present study and all investigated alleles had a prevalence of at least 4% (except rs45502302). Accordingly, frequent statistical problems of association studies, such as population stratification or genotyping error [44], are not relevant in our study.

## Conclusions

Taken together, our findings confirm a significant contribution of pharmacogenetic polymorphisms to the individual susceptibility of opioid addiction. In particular, we provide further support for a significant role of *GAL* and *OPRD1* in opioid dependency. In addition, our results indicate a potential contribution of *OPRM1* and *ABCB1* SNPs to the development of opioid addiction in the European population. The identified genetic susceptibility markers may contribute to the neurobiological understanding of addictive behaviour may help to develop novel therapeutic options. Thus, these variants should be addressed in further studies.

## Supporting Information

Protocol S1 (DOC)

Supporting Information S1 (DOC)
