# Supplementary material for: Association of Polymorphisms in Pharmacogenetic Candidate Genes (OPRD1, GAL, ABCB1, OPRM1) with Opioid Dependence in European Population: A Case-Control Study
Source: PLoS One. 2013 Sep 25;8(9):e75359. doi: 10.1371/journal.pone.0075359 (PMC3783401; doi:10.1371/journal.pone.0075359)
Supplement: Protocol S1 — (DOC) [file pone.0075359.s001.doc]

**Studienprotokoll**

**UNTERSUCHUNG DER GENETISCH BEDINGTEN VARIABILITÄT DES OPIOID- UND OPIATBEDARFS IM RAHMEN DES DROGENSUBSTITUTIONSPROGRAMMS**

**HÄUFIGKEITEN GENETISCHER POLYMORPHISMEN IN POPULATIONEN MIT UND OHNE OPIOIDABHÄNGIGKEITSERKRANKUNG**

**UND**

**UNTERSUCHUNG PHARMAKOGENETISCHE ASPEKTE BEI OPIOID- bzw. OPIATINDUZIERTEN DIREKTEN DROGENTODESFÄLLEN**

**Beate Beer^1^**

**Marion Pavlic^1^**

**Kathrin Libiseller^1^**

**Walther Parson^1^**

**Harald Niederstätter^1^**

**Salvatore Giacomuzzi^2^**

**Yvonne Riemer^2^**

**Stefanie Iwersen-Bergmann^3^**

**Hilke Andresen^3^**

**Herbert Oberacher^1^**

**^1^ Institut für Gerichtliche Medizin, Medizinische Universität Innsbruck**

**^2^ Universitätsklinik für Allgemeine Psychiatrie, Medizinische Universität Innsbruck**

**^3^Institut für Rechtsmedizin, Universitätsklinikum Hamburg-Eppendorf**

**SYNOPSIS**

**Sponsor**

Medizinische Universität Innsbruck

**Titel**

Untersuchung der genetisch bedingten Variabliltät des Opioid- und Opiatbedarfs im Rahmen des Drogensubstitutionsprogramms, Häufigkeiten genetischer Polymorphismen in Populationen mit und ohne Opioidabhängigkeitserkrankung und pharmakogenetische Aspekte des opioid- bzw. opiatbedingten direkten Drogentodes.

**Kurzbezeichnung**

Genetische Variabilität der Teilnehmer des Drogensubstitutionsprogramms.

**Zielpopulation**

Teilnehmer des Opioidsubstitutionsprogramms.

**Studiendesign**

unkontrollierte, explorative und einfachverblindete Studie mit kontrollierter explorativer Substudie 1 und unkontrollierter explorativer bizentrischer Substudie 2

**Studienziele**

Hauptstudie: 1) Untersuchung des Einflusses genetischer Polymorphismen auf die Variabilität des Opioid- bzw. Opiatbedarfs im Rahmen des Drogensubstitutionsprogramms. 2) Untersuchung des Einflusses genetischer Polymorphismen auf den Plasmaspiegel der Substitutionsopioide bzw. – opiate.

Substudie 1: Untersuchung der Häufigkeit bestimmter genetischer Polymorphismen in einer opioidabhängigen Population (anhand der Genotypisierungsdaten der Hauptstudie) und Vergleich dieser Häufigkeit mit einer Kontrollpopulation.

Substudie 2: Untersuchung pharmakologisch relevanter Genpolymorphismen bei direkten opiat-/opioid-induzierten Drogentodesfällen

**Zielgrößen:**

Hauptstudie: 1) Dosierung (mg/kg/d) 2) Opioid- bzw. Opiatplasmakonzentration (ng/ml).

Substudie 1: 1) Allelhäufigkeiten des OPRK1 36G>T Polymorphismus. 2) Allelhäufigkeiten genetischer Polymorphismen in den Genen OPRM1, OPRD1, ADRBK2, MDR1, STAT6, COMT, GAL, MC1R, 5HTR1A, DRD2, SCLO1B1, SCLO1A2, UGT2B7, UGT1A1 und UGT1A3.

Substudie 2: post-mortem Konzentration von Methadon/Heroin/Morphin und deren Metaboliten in peripherem Blut

**Patientenzahl:**

Hauptstudie: n = 300 Patienten der Ambulanz für Abhängigkeitserkrankungen.

Substudie 1: die Genotypisierungsdaten der Patienten werden von der Hauptstudie übernommen; zusätzlich wird eine Kontrollpopulation (n = 200) rekrutiert.

Substudie 2: 300 direkte opiat-/opioidinduzierte Drogentodesfälle

**Zeitplan:**

Patienten- bzw. Probandenrekrutierung: 2 Jahre. Sammelphase der Obduktionsasservate der Drogentodesfälle: 2 Jahre. Untersuchungsphase: 3 Jahre (beginnt während der Patientenrekrutierung). Follow-Up Phase: 1 Jahr. Vorgesehener Zeitpunkt des Studienbeginns bzw. -endes: WS 2008, WS 2013

**Einschlusskriterien**

Patienten: Diagnostizierte Opioidabhängigkeitserkrankung nach DSM-IV, Teilnehmer des Opioidsubstitutionsprogramms mit konstantem Dosierungsregime seit mindestens 8 Wochen, Alter >18 und <50 Jahre, Geschäftsfähigkeit bei Studieneinschluss, unterzeichnete Einverständniserklärung, inkludiert werden Männer und Frauen.

Kontrollpopulation (Substudie 1): keine Abhängigkeitserkrankung, Alter >18 und <50 Jahre, Geschäftsfähigkeit bei Studieneinschluss, unterzeichnete Einverständniserklärung, inkludiert werden Männer und Frauen.

Drogentodesfälle (Substudie 2): Vorliegen eines direkten Drogentodes, Methadon/Heroin/Morphin als toxikologische Hauptsubstanz in den Obduktionsasservaten, eventuell zusätzlich vorhandene Substanzen im peripheren Blut in einem nicht-toxischen Konzentrationsbereich, Lebensalter < 50 Jahre

**Ausschlusskriterien**

Patienten: keine Opioidabhängigkeitserkrankung nach DSM-IV, nachweislicher regelmäßiger Beikonsum von Kokain; wird erfasst durch das routinemäßig durchgeführte Harnscreening), Teilnahme an einer anderen klinischen Studie, diagnostizierte Leberzirrhose.

Kontrollpopulation (Substudie 1): fehlende Geschäftsfähigkeit bei Studieneinschluss

Drogentodesfälle (Substudie 2): indirekter Drogentod, zusätzliche Substanzen im peripheren Blut in toxischen Konzentrationen

**Ablauf der Studie**

Hauptstudie:

| **PATIENTENPOPULATION** | Woche 1 | Woche 4 | Woche 8 | Monat 1-6 | Monat 7-18 |
| --- | --- | --- | --- | --- | --- |
| Informationsgespräch | x |  |  |  |  |
| Mundhölenabstrich | x |  |  |  |  |
| Blutabnahme | x | x | x |  |  |
| Laboruntersuchungen |  |  |  | x |  |
| Auswertung |  |  |  |  | x |

Substudie 1:

Genotypisierungsdaten der Patienten werden von der Hauptstudie übernommen.

| **KONTROLLPOPULATION** | Woche 1 | Monat 1-6 | Monat 7-18 |
| --- | --- | --- | --- |
| Informationsgespräch | x |  |  |
| Mundhöhlenabstrich | x |  |  |
| Laboruntersuchungen |  | x |  |
| Auswertung |  |  | x |

Substudie 2:

- Sammeln der Obduktionsasservate der Drogentodesfälle über einen Zeitraum von 2 Jahren

- Beginn der toxikologischen und genetischen Zusatzuntersuchungen nach 1 Jahr Sammelphase.

**Studienbezogene Verfahren und Laboruntersuchungen**

Genetische Analysen: DNA-Extraktion, Polymerase Chain Reaction (PCR) und anschließende Genotypisierung mit einer auf Massenspektrometrie basierenden Technik (ICEMS).

Plasmaspiegelbestimmungen: Festphasenextraktion und anschließende quantitative Analyse mittels Flüssigkeitschromatographie-Massenspektrometrie, Gaschromatographie-Massen-spektrometrie

**Prüfmedikation für die Hauptstudie (= registrierte Arzneimittel)**

Methadon-, Buprenorphin- und Morphinpräparate (Methadonhydrochlorid, Subutex Sublingualtabletten, Suboxone Sublingualtabletten, Temgesic Sublingualtabletten, Substitol Retard Kapseln, Compensan Retard Filmtabletten, Kapanol CSR Kapseln)

**Behandlungsplan**

Durch die vorliegende Studie erfolgt keine Änderung am regulären Behandlungsprogramm des einzelnen Patienten der Ambulanz für Abhängigkeitserkrankungen. Die medikamentöse Therapie läuft für alle Studienteilnehmer in gewohnter Weise weiter und wird durch die (verblindete) Ärztin der Ambulanz für Abhängigkeitserkrankungen festgelegt.

Sonstige Informationen zur Studie:

Für die Substudie 2 sind **keine zusätzlichen bzw. studienspezifischen Maßnahmen bei der routinemäßig durchgeführten Obduktion erforderlich**. Die für die genetischen und toxikologischen Zusatzuntersuchungen herangezogenen Obduktionsproben werden von den im Rahmen der gerichtsmedizinischen Routine aufbewahrten Obduktionsasservaten entnommen.

**1 EINFÜHRUNG**

**1.1 Die Opioidpharmakogenetik in der Substitutionstherapie (Hauptstudie)**

Individuell unterschiedliche Wirkungen einer definierten Arzneimitteldosis sind im klinischen Alltag keine Seltenheit. Nicht nur das Versagen der Therapie stellt für einigen Patienten und Ärzte ein Problem dar, sondern auch schwere bis hin zu fatal verlaufende Arzneimittelnebenwirkungen. Viele dieser abnormen Reaktionen sind genetisch determiniert. Durch Polymorphismen in Genen die für metabolisierende Enzyme, Transporterproteine oder spezifische Rezeptoren kodieren, können Plasmaspiegel und die pharmakologische Wirkung bestimmter Substanzen hoch variabel ausfallen.[[1]](#footnote-2) Genetische Polymorphismen und dadurch beeinflusste Arzneimittelwirkungen scheinen auch besonders bei Substanzen aus der Opioidklasse eine bedeutende Rolle zu spielen.[[2]](#footnote-3)

In Behandlungsprogrammen für opioidabhängige Personen werden in Österreich die langwirksamen Opioide bzw. Opiate Methadon, Buprenorphin und retardiertes Morphin als orale Substitutionsmittel eingesetzt. Obwohl auch diese Substanzen zu einer Abhängigkeit führen, ermöglichen sie im Rahmen von richtlinienkonformen Substitutionsprogrammen eine physische und psychosoziale Stabilisation der Teilnehmer. Drogenassoziierte Kriminalität und Komplikationen durch intravenösen Drogengebrauch wie z.B. Infektionen (HIV, Hepatitis B und C u.a.) können so erfolgreich reduziert werden.[[3]](#footnote-4),[[4]](#footnote-5),[[5]](#footnote-6)

Im Jahr 2006 waren in Österreich 8120 Personen als Teilnehmer des Opioid-Substitutionsprogramm gemeldet. Die jährlich steigende Zahl der Meldungen deutet auf eine zunehmende Akzeptanz und Inanspruchnahme dieses Therapieprogramms hin. Es hat sich mittlerweile zahlenmäßig zur wichtigsten und auch kosteneffektivsten Behandlungsstrategie für Opioidabhängigkeit entwickelt.Error: Reference source not found,[[6]](#footnote-7) Derzeitige Empfehlungen weisen auf eine Langzeiterhaltungstherapie im Sinne einer schrittweisen und langsamen Reduktion des verschriebenen Opioids erst nach einer langjährigen Behandlung (mehrere Jahre bis Jahrzehnte) hin. Kurzzeitinterventionen und vorzeitige Reduktionsmethoden haben sich als nicht zielführend herausgestelltError: Reference source not found.

Das jeweilige Dosierungsregime der Ersatzsubstanzen wird individuell an den Bedarf des jeweiligen Patienten angepasst. Wird die regelmäßige Dosis zu hoch gewählt, kann dies zu als unangenehm empfundenen und gefährlichen opioidtoxischen Symptomen wie z.B. Schwindel, Sedierung, Sopor oder Atemdepression führen[[7]](#footnote-8). Eine zu niedrig gewählte Dosis führt zum Weiterbestehen der Entzugssymptomatik und des sog. „Opioidhungers“ („craving“). Die Folge kann einerseits ein Abbruch der Therapie[[8]](#footnote-9), andererseits der illegale Beikonsum zentral aktiver Substanzen, darunter auch Heroin, sein. Polytoxikomane Konsummuster, bei denen die sich teilweise potenzierende Wirkung unterschiedlicher Substanzen schwer kontrollierbar ist, stellen die Hauptursache der suchtgiftbezogenen Todesfälle in Österreich dar. Um die Anzahl von Rückfällen, Kriminalitätsdelikten oder suchtgiftbezogenen Todesfällen möglichst klein zu halten, ist ein optimiertes Dosierungsregime für die langfristige Drogenersatztherapie von enormer WichtigkeitError: Reference source not found.

Die individuellen Dosierungsshemata sind hoch variabel und schwanken zwischen 20-150 mg pro Tag bei Methadon, 2-24 mg pro Tag bei Buprenorphin[[9]](#footnote-10) und 120-1200 mg bei Morphin. Diese beträchtlichen Schwankungsbreiten sind durch unterschiedliche Toleranzentwicklung aufgrund ungleicher Konsumgewohnheiten bzw. verschieden langer Abhängigkeit nicht vollständig erklärbar. Genetische, die Pharmakologie der Opioide beeinflussende Polymorphismen, scheinen eine bedeutende Rolle zu spielen.[[10]](#footnote-11)

Für Morphin konnte im Rahmen der Schmerztherapie gezeigt werden, dass spezielle allelische Varianten des µ-Opioidrezeptor-Gens (OPRM1) die analgetische Potenz und dadurch die Dosierungsbedürfnisse dieses Schmerzmittels beeinflussen. Des Weiteren konnten Polymorphismen im Multidrug Resistance Gen (MDR1)Error: Reference source not found,[[11]](#footnote-12) und im Katechol-O-Methyltransferase Gen (COMT, EC2.1.1.6) als signifikante Einflussfaktoren der Therapieeffizienz und der Dosierung von Morphin identifiziert werden.

Obwohl ein optimiertes und individuelles Dosierungsregime nicht nur im Schmerzmanagement, sondern auch besonders im Drogensubstitutionsprogramm von großer Wichtigkeit für den Erfolg der Therapie ist, sind pharmakogenetische Studien bezüglich des individuellen Dosierungsbedarfs an Substitutionsopioiden bzw. –opiaten für Methadon bisher kaum[[12]](#footnote-13), für Buprenorphin und Morphin nicht vorhanden.

Für die vorliegende Studie ein Kollektiv an Genen ausgewählt, die für die Opioid- bzw. Opiatwirkung pharmakologisch relevant sind. Polymorphismen in den angeführten Genen, entweder einzeln oder im Zusammenspiel, könnten einen bedeutenden Einfluss auf die pharmakologische Wirkung der Substitutionsopioide ausüben.

OPRM1

Eine Reihe von Polymorphismen des µ-Opioidrezeptor-Gens wurden beschrieben. Für den funktionell relevanten 118A>G Polymorphismus konnte gezeigt werden, dass dieser die analgetische Potenz einer definierten Morphindosis beeinflusst[[13]](#footnote-14). Onkologische Patienten, die Träger des 118A>G Polymorphismus sind, brauchen im Rahmen der Schmerztherapie eine signifikant höhere Dosis an Morphin als Träger des Wildtyps[[14]](#footnote-15). Auch die zentralnervöse Wirkung von Methadon und Buprenorphin, die ebenfalls an den µ-Opioidrezeptor binden, könnte durch diesen Polymorphismus verändert sein.

OPRK1

Genetische Polymorphismen des κ-Opioidrezeptors (OPRK1) wurden mit einer signifikant erhöhten Vulnerabilität für Suchtverhalten in Verbindung gebracht. Insbesondere der nichtfunktionelle Polymorphismus 36G>T, der vermutlich die Transkription oder Stabilität der mRNA beeinflusst, wurde häufiger bei heroinabhängigen Personen als in einer Vergleichspopulation gefunden[[15]](#footnote-16),[[16]](#footnote-17). Die Auswirkungen dieser genetischen Variation auf die Dosierungsschemata von Methadon, Buprenorphin oder retardiertem Morphin wurden bisher nicht untersucht.

STAT6

Der Transkriptionsfaktor STAT6 bindet an die Promoterregion des µ-Opioidrezeptors und ist dadurch an der Regulation der Genexpression des Rezeptors beteiligt. Es konnte gezeigt werden dass Träger eines bestimmten STAT6 Allels unter Morphinmedikation signifikant häufiger unter Intoleranzreaktionen leiden als Träger des Wildtypallels[[17]](#footnote-18). Dies könnte auch für die Opioide Methadon und/oder Buprenorphin bedeutend sein und wertvolle Hinweise auf deren Verträglichkeit liefern.

COMT

Die Katechol-O-Methyltransferase (COMT, EC 2.1.1.6) ist am Abbau der biogenen Amine Dopamin, Noradrenalin und Adrenalin beteiligt[[18]](#footnote-19). Die Aktivität dieses Enzyms wird signifikant durch den Val158Met Polymorphismus beeinflusst. Eine Assoziation dieses genetischen Polymorphismus mit der morphininduzierter Analgesie bzw. mit Morphin-Dosisbedürfnissen liegt vor. Ursächlich wird eine indirekte Korrelation zwischen der COMT-Enzymaktivität und der Opioidrezeptor-Dichte angenommen[[19]](#footnote-20).

MDR1

Das P-Glykoprotein, kodiert durch das Multidrug Resistance Gen (MDR1), dient als multispezifische, u.a. an der Blut-Hirn-Schranke lokalisierte Effluxpumpe. Auch innerhalb dieses Gens finden sich diverse Polymorphismen, die maßgeblich die Bioverfügbarkeit zentral wirksamer Substanzen jenseits der Blut-Hirn-Schranke beeinflussen können. Eine positive Korrelation zwischen genetischen MDR1-Varianten und dem Methadondosierungsregime konnte dargelegt werden[[20]](#footnote-21).

UGT1A1, UGT1A3

Die Gene UGT1A1 und UGT1A3 kodieren UDP-Glukuronyltransferasen mit hoher Substratspezifität für Buprenorphin. Die Relevanz von Polymorphismen in diesen Genen für die Aktivität der UDP-Glukuronyltransferasen wurde zum Beispiel schon im Zusammenhang mit der antineoplastischen Substanz Irinotecan in vivo[[21]](#footnote-22) und für Estron in vitro[[22]](#footnote-23) dokumentiert, wurde jedoch noch nicht für Buprenorphin untersucht.

MC1R Gen

Mogil JS et al. konnten kürzlich sowohl im Tierversuch als auch im Rahmen einer klinischen Studie zeigen, dass Individuen, die Träger einer bestimmten “loss-of-function“ Mutation im Melanocortin-1 Rezeptorten Gen sind, stärker auf die analgetische Wirkung von Morphin-6-Glucuronid ansprechen als Träger des Wildtypgens.[[23]](#footnote-24) Grundsätzlich könnte dieser SNP auch für den pharmakodynamischen Efffekt anderer Opiate/Opioide von Bedeutung sein.

5-HT1A Gen

Varianten des 5-HT1A Rezeptor Gens scheinen eine immer größer werdende Rolle im Zusammenhang mit psychiatrischen Krankheitsbildern wie z.B. Depressionen, Angststörungen und Psychosen zu spielen.[[24]](#footnote-25) Die Rolle von genetischen 5-HT1A-Polymorphismen im Zusammenhang mit Abhängigkeitserkrankungen und süchtigem Verhalten wurde bisher noch nicht untersucht.

Durch Prüfung bereits beschriebener Varianten der angeführten Gene hinsichtlich ihrer Bedeutung für die Substitutionstherapie könnten wertvolle Informationen für die Drogenersatztherapie entstehen. Anhand eines pharmakogenetischen Profils könnten Patienten spezifischer auf eines der zur Verfügung stehenden Opioide eingestellt werden. Derartige Polymorphismen könnten sich nicht nur auf die individuelle Dosierung, sondern auch auf die Wahl des Substitutionsopioids auswirken, da die verschiedenen Substanzen unterschiedliche Rezeptorbindungsprofile aufweisen und von unterschiedlichen Enzymen metabolisiert werden. Des Weiteren könnte diese Studie auch wertvolle Implikationen für die Opioidschmerztherapie mit sich bringen.

**1.2 Gibt es einen Einfluss von genetischen Polymorphismen auf das individuelle Suchtverhalten (Substudie 1)?**

Es gibt Hinweise, dass genetische Polymorphismen nicht nur an der pharmakologischen Wirkung von Opiaten- und Opioiden mitbeteiligt sind, sondern auch Einfluss auf das individuelle Suchtverhalten nehmen. Gerra *et al*. konnten kürzlich zeigen, dass ein spezieller Polymorphismus im Exon 2 des κ-Opioidrezeptor-Gens des bei opioidabhängigen Personen signifikant häufiger zu finden ist als in einer gesunden Kontrollpopulation (Allelhäufigkeit 10,4 % versus 4,3 %)Error: Reference source not found. Auch der OPRM1 118A>G Polymorphismus soll häufiger bei Personen mit Opioidabhängigkeitserkrankung vorkommen bzw. mit einer erhöhten Vulnerablität für Suchtverhalten einhergehen[[25]](#footnote-26). Generell wird der Beitrag von genetischen Faktoren zum Ausmaß des individuellen Suchtverhalten moderat bis groß eingeschätzt[[26]](#footnote-27).

Aus diesem Grund möchten wir im Rahmen der vorliegenden Studie die genannten Allelhäufigkeiten des OPRK1- und OPRM1-Gens für eine mitteleuropäische Population überprüfen und auch weitere Allelverteilungen (z.B. des STAT6 und COMT-Gens) beleuchten. Im Rahmen einer kontrollierte Substudie sollen die durch die Hauptstudie gewonnenen Genotypisierungsdaten bzw. Allelhäufigkeiten einer vergleichbaren, nicht opioidabhängigen Kontrollpopulation gegenübergestellt werden.

**1.3 Pharmakogenetische Aspekte des opioidinduzierten Drogentods (Substudie 2)**

Generell wird mit dem Begriff Drogentod jene Form des Ablebens bezeichnet, die durch den Konsum illegaler Substanzen verursacht wird. Man unterscheidet hierbei die direkten Drogentodesfälle infolge beabsichtigter und unbeabsichtigter Überdosierung von Suchtmitteln von den indirekten Drogentodesfällen, die durch die Folgen eines langzeitigen Drogenmissbrauchs verursacht werden (z.B. Infektionen, toxische Organschäden) und kausal nicht in einem direkten Zusammenhang mit einem aktuellen Suchtgiftkonsum stehen.[[27]](#footnote-28) Durchschnittlich werden in Europa jährlich etwa 7000 bis 8000 direkte Drogentodesfälle gemeldet, wobei diese Zahl als Mindestschätzung gesehen werden muss, da nicht alle Fälle gemeldet werden. Weitaus führend sind dabei jene Todesfälle, die mit dem Konsum von Opiaten bzw. Opioiden in Zusammenhang stehen.[[28]](#footnote-29) So wurden etwa im Jahr 2007 bei 97 % aller suchtgiftbezogenen Todesfälle in Österreich Opiate nachgewiesen.^27^

Die Frage, ob Überdosierungen von Suchtmitteln in suizidaler Absicht oder akzidentiell zustande kommen, ist im Einzelfall schwierig zu beantworten. Man geht jedoch davon aus, dass ein Großteil der Drogentodesfälle von akzidentieller Natur sind.[[29]](#footnote-30),[[30]](#footnote-31) Als Risikofaktoren für akzidentielle Überdosierungen werden neben einem Toleranzverlust nach Entzug bzw. Haft oder dem veränderten Reinheitsgrad der Schwarzmarktsubstanzen auch individuelle (genetische) Faktoren, wie z.B. eine „vorbestehende Sensibilität“ gegenüber der Opiatwirkung angegeben.[[31]](#footnote-32) Bislang wurden unseres Wissens jedoch noch keine pharmakogenetischen Untersuchungen in Bezug auf den opiat-/opioidbedingten Drogentod publiziert.

Eine potentielle Relevanz der Opioidpharmakogenetik für den suchtgiftbezogenen Tod ergibt sich aus der Tatsache, dass die bei Drogentoten gefundenen Opioid- bzw. Opiatblutspiegel interindividuell höchst unterschiedlich sind. Zudem werden bei opioidinduzierten Todesfällen Blutkonzentrationen gefunden, die auch bei Lebenden, z.B. Teilnehmern des Drogensubstitutionsprogramms, beobachtet werden können, ohne dass diese mit relevanten toxikologischen Beeinträchtigungen einhergehen.[[32]](#footnote-33),[[33]](#footnote-34),[[34]](#footnote-35) Ein potentiell letaler Opioidblutspiegel kann somit weniger an einer bestimmten Zahl, sondern vielmehr an individuellen Faktoren festgemacht werden. Nur teilweise kann diese variable Verträglichkeit durch interindividuell unterschiedliche Gewöhnungs- bzw. Entwöhnungseffekte oder pharmakologische Interaktionen mit zusätzlich konsumierten Substanzen erklärt werden. Eine Untersuchung der Todesfälle hinsichtlich pharmakogenetischer Aspekte erscheint daher sinnvoll und potentiell relevant.

Das primäre Ziel der Substudie 2 ist daher, bestimmte Polymorphismen in pharmakologisch relevanten Genen, die u.a. für metabolisierende Enzyme, Transporterproteine und Rezeptoren kodieren, in einem Studienkollektiv aus Drogentoten zu untersuchen. Im Speziellen sollen Polymorphismen folgender Gene untersucht werden:

- OPRM (µ-opioid receptor gene)
- OPRD (δ-opioid receptor gene)
- OPRK (κ-opioid receptor gene)
- COMT (catechol-O-methyltransferase)
- STAT6 (signal transducer and activator of transcription 6)
- MDR1 (ATP-binding cassette, subfamiliy B, member 1)
- UGT1A1 (UDP-glucosyltransferase 1 family, polypeptide A1)
- DRD2 (dopamine receptor D2)
- GAL (galanine)
- SLCO1B1 (solute carrier organic anion transporter family, member 1B1)
- SLCO1A2 (solute carrier organic anion transporter family, member 1A2)
- MC1R (melanocortin 1 receptor)
- UGT2B7 (uridine diphosphate glycosyltransferase 2 famliy, member B7)
- 5-HTR1A (5α hydroxytryptamine receptor 1 A)
- GRK3 (beta-adrenic receptor kinase 2)

Eine Untersuchung der pharmakokinetischen und pharmakodynamischen Merkmale des opiatindzierten Drogentodes und der inter-individuellen Unterschiede z.B. in Abhängigkeit von genetischen Polymorphismen, kann nicht nur zu einem **tieferen Verständnis der individuellen Risiken für den Drogentod**, sondern auch **wertvolle Implikationen für die Schmerztherapie und Drogensubstitutionstherapie** mit sich bringen.

**HAUPTSTUDIE:**

**UNTERSUCHUNG DER GENETISCH BEDINGTEN VARIABILITÄT DES OPIOID- UND OPIATBEDARFS IM RAHMEN DES DROGENSUBSTITUTIONSPROGRAMMS**

**2. Ziele des Hauptstudie**

**2.1. Primäres Studienziel**

Das primäre Ziel der Hauptstudie ist, den Einfluss genetischer Faktoren auf die Variabilität des Opioid- und Opiatbedarfs zu untersuchen. Bei Teilnehmern des Drogensubstitutionsprogramms der Ambulanz für Abhängigkeitserkrankungen („Drogenambulanz“ der Universitätsklinik für Psychiatrie, MUI) sollen für die Drogensubstitutionstherapie pharmakologisch relevante Polymorphismen in den Genen OPRM1, OPRK1, MDR1, COMT, STAT6, MC1R, 5HT1A, UGT1A1 und UGT1A3 identifiziert und mit dem jeweils erforderlichen Dosierungsschema des verschriebenen Opioids bzw. Opiats korreliert werden (Genotyp-Phänotyp-Korrelation, s. Übersicht S.11).

Unseres Wissens wurden bisher bezüglich der Dosierungsschemata von Buprenorphin und retardiertem Morphin im Rahmen der Opioidersatztherapie noch keine pharmakogenetischen Assoziationen durchgeführt. Über die Entwicklung eines tieferen Verständnisses für pathophysiologische und pharmakogenetische Vorgänge hinaus könnte durch Identifikation pharmakologisch relevanter Polymorphismen Vorhersagen über die individuelle Wirkung eines Substitutionsopioids gemacht werden. Dies würde in Zukunft eine näher an den individuellen Bedarf des Patienten angepasste [Dosierung](http://de.wikipedia.org/wiki/Dosierung) ermöglichen bzw. relative Über- oder Unterdosierungen, die zu Therapieabbruch oder gefährlichem Beikonsum zentral aktiver Substanzen führen können, vermeiden.

**2.2 Sekundäre Studienziele**

Des Weiteren sollen durch diese Studie wertvolle Informationen über die jeweiligen Plasmaspiegel der Substitutionssubstanzen und deren (genetisch bedingte) Variabilität zwischen Patienten mit identischem Dosierungsschema gewonnen werden. Auch gegebenenfalls vorhandene geschlechtsspezifische Unterschiede in Dosierungsregime und Plasmaspiegel sollen im Rahmen dieser Studie erfasst werden.

**Übersicht: Primäre und Sekundäre Studienziele**

**GENETISCHE POLYMORPHISMEN ⬄ ERFORDERLICHE OPIOID- BZW. OPIATDOSIS (mg/kg/d)**

**GENETISCHE POLYMORPHISMEN ⬄ PLASMASPIEGEL DER OPIOIDE BZW. OPIATE (ng/ml)**

**BEI PATIENTEN MIT IDENTISCHER DOSIS**

**PLASMASPIEGEL (ng/ml) ⬄ OPIAT- BZW. OPIOIDDOSIS**

**3. Beschreibung des Hauptstudie**

**3.1. Studiendesign**

Es handelt sich bei dieser Studie um eine unkontrollierte, explorative und verblindete Studie. Die Einstellung der Dosierung erfolgt durch eine verblindete Person, d.h. der zuständigen Ärztin werden die Ergebnisse der Genotypisierung nicht mitgeteilt. Dadurch wird eine mögliche Bias durch die Dosierungseinstellung vermieden. Da die Einstellung auf das jeweilige Opiat bzw. Opioid nur durch eine Person stattfindet, ist kein Untersuchereffekt zu erwarten.

**3.2 Primäre Hypothese**

Das Vorkommen bestimmter genetischer Polymorphismen im Genom beeinflusst signifikant den individuellen Bedarf an Opiaten bzw. Opioiden im Rahmen der Substitutionstherapie.

**3.3 Sekundäre Hypothese**

Das Vorkommen bestimmter genetischer Polymorphismen im Genom eines Patienten beeinflusst signifikant den individuellen Plasmaspiegel von Opiaten bzw. Opioiden im Rahmen der Substitutionstherapie.

**3.4 Patientenzahlen**

Das Patientenkollektiv der Ambulanz für Abhängigkeitserkrankungen der Universitätsklinik für Psychiatrie (Medizinische Universität Innsbruck) besteht derzeit aus rund 800 Patienten. Voraussichtlich werden davon ca. 300 Patienten in die Studie eingeschlossen. Von diesen 300 Patienten werden sich in etwa 120 Patienten in Gruppe 1 (Methadongruppe), 100 Patienten in Gruppe 2 (Buprenorphingruppe) und 80 Patienten in Gruppe 3 (Morphingruppe) befinden.

**3.5 Zeitplan**

- Patientenrekrutierung: 2 Jahre
- Untersuchungsphase: 3 Jahre (beginnt während der Patientenrekrutierung)
- Follow-Up Phase 1 Jahr
- Vorgesehener Zeitpunkt des Studienbeginns: WS 2008
- Voraussichtlicher Zeitpunkt des Studienendes: WS 2012

Über- oder Unterschreitungen des Zeitplans werden keinen Einfluss auf die Studie nehmen.

**4. Studienpopulation**

Da im Rahmen des Substitutionsprogramms drei verschiedene Opioide bzw. Opiate verabreicht werden, wird für die Hauptstudie die Studienpopulation voraussichtlich in drei Gruppen eingeteilt.

**4.1 Einschlusskriterien Patientengruppe 1 (Methadongruppe)**

- diagnostizierte Opioidabhängigkeitserkrankung nach DSM-IV Kriterien
- täglicher Heroin-/Morphin-Missbrauch seit mind. 2 Jahren in der Vorgeschichte
- Teilnehmer des Opioidsubstitutionsprogramms mit konstantem Dosierungsregime seit mindestens 8 Wochen
- Methadon als orales Substitutionsmittel
- Alter >18 Jahre, < 50 Jahre
- Geschäftsfähigkeit des Patienten bei Studieneinschluss
- unterzeichnete Einverständniserklärung
- inkludiert werden Männer und Frauen

**4.2 Einschlusskriterien Patientengruppe 2 (Buprenorphingruppe)**

- diagnostizierte Opioidabhängigkeitserkrankung nach DSM-IV Kriterien
- täglicher Heroin-/Morphin-Missbrauch seit mind. 2 Jahren in der Vorgeschichte
- Teilnehmer des Opioidsubstitutionsprogramms mit konstantem Dosierungsregime seit mindestens 8 Wochen
- Buprenorphin als orales Substitutionsmittel
- Alter >18 Jahre, < 50 Jahre
- Geschäftsfähigkeit des Patienten bei Studieneinschluss
- unterzeichnete Einverständniserklärung
- inkludiert werden Männer und Frauen

**4.3 Einschlusskriterien Patientengruppe 3 (Morphingruppe)**

- diagnostizierte Opioidabhängigkeitserkrankung nach DSM-IV Kriterien
- täglicher Heroin-/Morphin-Missbrauch seit mind. 2 Jahren in der Vorgeschichte
- Teilnehmer des Opioidsubstitutionsprogramms mit konstantem Dosierungsregime seit mindestens 8 Wochen
- retardiertes Morphin als orales Substitutionsmittel
- Alter >18 Jahre, < 50 Jahre
- Geschäftsfähigkeit des Patienten bei Studieneinschluss
- unterzeichnete Einverständniserklärung
- inkludiert werden Männer und Frauen

**4.4 Ausschlusskriterien Patientengruppe 1, 2 und 3**

- keine Opioidabhängigkeitserkrankung nach ICD10-F11.2
- nachweislicher (Harnscreening) regelmäßiger Beikonsum von Kokain
- Teilnahme an einer anderen klinischen Studie
- diagnostizierte Leberzirrhose
- bestehende oder geplante Schwangerschaft
  1. **Ausscheiden von Patienten während der Studie (Drop-out)**

Die Teilnahme an der Studie erfolgt freiwillig. Die Patienten können jederzeit ohne Angabe von Gründen aus der Studie ausscheiden. Die Ablehnung der Teilnahme, das Zurückziehen der Einverständniserklärung oder ein Ausscheiden während der Studie hat keine nachteiligen Folgen für die weitere medizinische Betreuung.

- 1. **Ausschluss von Patienten aus der Studie**

Ebenso kann der Prüfer der Studie Probanden jederzeit aus folgenden Gründen aus der Studie ausschließen:

- Abbruch der Substitutionstherapie
- regulärer Beikonsum von Kokain

**A5 Prüfmedikation**

Im Rahmen dieser Studie werden keine Änderungen am individuellen Dosierungsregime des einzelnen Patienten vorgenommen. Die Behandlung läuft für alle Studienteilnehmer in gewohnter Weise weiter. Änderungen an der Therapie dürfen nur durch die zuständige (verblindete) Ärztin der Drogenambulanz im Rahmen des regulären Behandlungsprogramms erfolgen.

**A5.1 Beschreibung der Medikation**

**Methadon**

Präparat

- Methadonhydrochlorid

Reinheitsgrad > 99 %

Hersteller: Gatt-Koller Pharmazeutika

Die gebrauchsfertige dosisangepasste orale Schlucklösung (mit Zuckersirup) wird täglich für den einzelnen Patienten vom zuständigen Apotheker zubereitet.

Bereitstellung:

Durch die jeweilige Apotheke. Abgabe nur auf Suchtgiftrezept (Ausstellung durch die Ärztin der Ambulanz für Abhängigkeitserkrankungen).

Lagerung, Haltbarkeit, Aufbewahrungshinweise, Vorsichtsmaßnahmen s. beiliegende Fachinformationen[[35]](#footnote-36).

**Buprenorphin**

Präparate

- Subutex 2 mg und 8 mg Sublingualtabletten

enthält 2 mg bzw. 8 mg Buprenorphin (als Hydrochlorid)

Hilfsstoffe: Lactose Monohydrat, Mannitol, Maisstärke, Providon K30, Zitronensäure, Natriumzitrat, Magnesiumstearat

Hersteller: AESCA Pharma GmbH

- Suboxone 2 mg (+ 0,5 mg Naloxon) und 8 mg (+ 2 mg Naloxon) Sublingualtabletten

enthält 2 mg bzw. 8 mg Buprenorphin (als Hydrochlorid) und 0,5 mg bzw. 2 mg Naloxon (als Naloxonhycrochlorid-Dihydrat)

Hilfsstoffe: Lactose Monohydrat, Mannitol, Maisstärke, Providon K30, Zitronensäure, Natriumzitrat, Magnesiumstearat, Acesulfam-Kalium, natürliches Zitronen- und Limonenaroma

Zulassungsinhaber: SP Europe

- Temgesic 0,2 mg und 0,4 mg Sublingualtabletten

enthält 0,2 mg bzw. 0,4 mg Buprenorphin (als Hydrochlorid)

Hilfsstoffe: Lactose Monohydrat, Mannitol, Maisstärke, Providon K30, wasserfreie Zitronensäure, Natriumzitrat, Magnesiumstearat

Hersteller: AESCA Pharma GmbH

Bereitstellung:

Durch die jeweilige Apotheke. Abgabe nur auf Suchtgiftrezept (Ausstellung durch die Ärztin der Ambulanz für Abhängigkeitserkrankungen).

Lagerung, Haltbarkeit, Aufbewahrungshinweise, Vorsichtsmaßnahmen s. beiliegende Fachinformationen.Error: Reference source not found

**Morphin**

Präparate

- Substitol Retard 120 mg und 200 mg Kapseln

enthält 120 mg bzw. 200 mg Morphinsulfat-pentahydrat entsprechend 90 mg bzw. 150 mg Morphin

Hilfsstoffe: hydriertes Pflanzenöl, Macrogol 6000, Talkum, Magnesiumstearat,

Gelatine, Farbstoffe E 132, E 172, E 173, Schellack, Sojalecithin, Dimethylpolysiloxan

Hersteller: Mundipharma Ges.m.b.H

- Compensan Retard 100 mg, 200 mg und 300 mg Filmtabletten

enthält 100 mg, 200 mg bzw. 300 mg Morphin hydrochlorid trihydrat, entsprechend 75,95 mg, 151,9 mg bzw. 227,85 mg Morphin

Hilfsstoffe: Lactose Monohydrat, Polyacrylat Dispersion 30%, Methacrylsäure-Ethylacrylat Copolymer (1:1), Ammonium Methacrylat Copolymerisat Typ B, Hypromellose 4000, Magnesiumstearat, Macrogol 6000, Hypromellose 5, Titandioxid E171, Lebensmittelfarbstoffe: E104, E110

Hersteller: Lannacher Heilmittel Ges.m.b.H

- Kapanol CSR 50 mg und 100 mg Kapseln

enthält 50 mg bzw 100 mg Morphinsulfat-Pentahydrat, entsprechend 37,5 mg bzw. 75 mg Morphin

Hilfsstoffe: Zuckerpellets (Saccharose-Maisstärke), Hypromellose, Ethylcellulose, Methacrylsäurecopolymer Typ C, Polyethylenglykol 6000, Diethylphthalat, Talkum, Gelatine, E 172, Schellack, Propylenglykol

Hersteller: GlaxoSmithKline Pharma GmbH

Bereitstellung:

Durch die jeweilige Apotheke. Abgabe nur auf Suchtgiftrezept (Ausstellung durch die Ärztin der Ambulanz für Abhängigkeitserkrankungen).

Lagerung, Haltbarkeit, Aufbewahrungshinweise, Vorsichtsmaßnahmen s. beiliegende Fach-informationen.Error: Reference source not found

**A5.2 Verzeichnis der Neben- und Wechselwirkungen**

Siehe die dem Antrag beigelegte Fachinformationen.Error: Reference source not found

**A5.3 Behandlungsschema**

Das Behandlungsschema wird durch die behandelnde Ärztin der Ambulanz für Abhängigkeitserkrankungen (verblindet) festgelegt. Die Einnahme des Präparats durch den Patienten erfolgt 1-mal täglich, jeweils zur selben Uhrzeit.

**A5.4 Verblindung**

Die medikamentöse Behandlung der Teilnehmer des Drogenersatzprogramms bzw. die Einstellung der Patienten auf eine entsprechende Dosierung erfolgt durch eine verblindete Person, d.h. die zuständige Ärztin der Ambulanz für Abhängigkeitserkrankungen wird nicht über die Genotypisierungsergebnisse in Kenntnis gesetzt (Einfachverblindung).

**5 Ablauf der Hauptstudie**

Im Rahmen dieser Studie werden keine Änderungen am persönlichen Dosierungsregime des einzelnen Patienten vorgenommen. Die Behandlung läuft für alle Studienteilnehmer in gewohnter Weise weiter. Änderungen an der Therapie dürfen nur durch die zuständige (verblindete) Ärztin der Ambulanz für Abhängigkeitserkrankungen im Rahmen des regulären Behandlungsprogramms erfolgen.

**5.1 Visite 1**

Die Rekrutierung der Patienten erfolgt im Rahmen der regulären Sprechstunde der Ambulanz für Abhängigkeitserkrankungen der Universitätsklinik für Psychiatrie (MUI). Vor Studieneinschluss erfolgt ein ausführliches Informationsgespräch mit detaillierter Beantwortung allfälliger Fragen. Anschließend wird die schriftliche „Patienteninformation und Einwilligungserklärung zur Teilnahme an einer klinischen Studie“ vorgelegt.

Bei Einwilligung zur Teilnahme und dem Vorliegen einer unterzeichneten Einverständniserklärung werden für die genetischen Untersuchungen mit Filzkämmen drei Mundhöhlenabstriche (MHA) zur Gewinnung der DNA abgenommen. Dies erfolgt einmalig und ist völlig schmerzfrei und risikolos.

Im Zuge der Visite 1 erfolgt außerdem eine Abnahme von venösem Blut (1 x 10 ml) zur Bestimmung des aktuellen Plasmaspiegels des jeweiligen Substitutionsopioids. Dabei wird darauf geachtet, dass die Blutabnahme jeweils in der sog. Steady-State-Phase des Plasmaspiegelverlaufs erfolgt. Die Zeitspanne zwischen letzter Einnahme des Opioids bzw. Opiats und der Blutabnahme wird genau dokumentiert.

Außerdem wird im Rahmen der Visite 1 ein Termin für Visite 2 und Visite 3 vereinbart. Auch diese Termine werden so organisiert, dass die Zeitdifferenzen zwischen letzter Einnahme des Opioids bzw. Opiats und Blutabnahme für alle Patienten in einem vergleichbaren Zeitrahmen liegen.

**5.3 Visite 2**

Visite 3 findet vier Wochen nach Visite 1 statt und dient der erneuten Blutabnahme (1 x 10 ml) zur Serumspiegelkontrolle. Die Termine werden wie bei Visite 2 organisiert. Der Zeitpunkt der Blutabnahme bzw. der letzten Opioideinnahme werden genau dokumentiert.

**5.4 Visite 3**

Visite 3 findet acht Wochen nach Visite 1 statt und verläuft analog zur Visite 2

**5.5 Laboruntersuchungen**

Die genetischen Analysen und die Bestimmungen der Plasmakonzentrationen, werden am Institut für Gerichtliche Medizin durchgeführt. Die Spiegelbestimmungen der Substanzen erfolgen nach Festphasenextraktion mit geeigneten analytischen Methoden (Flüssigkeitschromatographie-Massenspektrometrie, Gaschromatographie-Massenspektro-metrie). Die genetischen Analysen erfolgen nach DNA-Extraktion mittels Polymerase Chain Reaction (PCR) und anschließender Genotypisierung mit einer auf Massenspektrometrie basierenden Technik (ICEMS).

**Zeitplan für die jeweiligen Probanden der Gruppe 1, Gruppe 2 und Gruppe 3:**

|  | Woche 1 | Woche 4 | Woche 8 | Monat 1-6 | Monat 7-18 |
| --- | --- | --- | --- | --- | --- |
| Informationsgespräch | x |  |  |  |  |
| Mundhölenabstrich | x |  |  |  |  |
| Blutabnahme | x | x | x |  |  |
| Labor |  |  |  | x |  |
| Auswertung |  |  |  |  | x |

**5.6 Weiterbehandlung**

Die Therapie läuft für den einzelnen Patienten während und nach Abschluss der Studie regulär weiter.

**5.7 Ausscheiden von Patienten**

Die Probanden können jederzeit ohne Angabe von Gründen aus der Studie ausscheiden. Das Zurückziehen der Einverständniserklärung bzw. ein Ausscheiden während der Studie hat keine nachteiligen Folgen für die weitere medizinische Betreuung.

**5.8 Ausschluss von Probanden aus der Studie**

Ebenso kann der Prüfer der Studie Probanden jederzeit aus folgenden Gründen aus der Studie ausschließen:

- Abbruch der Substitutionstherapie
- chronischer, nachweislicher Beikonsum andere Opioide, Kokain oder Benzodiazepinen (wird durch das routinemäßig durchgeführte Harnscreening erfasst)

**A6 Pharmakovigilanz**

**A6.1 Unerwünschte Arzneimittelwirkungen, Meldeprozeduren**

Unerwünschte Arzneimittelwirkungen werden von der behandelnden Ärztin der Ambulanz für Abhängigkeitserkrankungen im Rahmen der regulären Sprechstunde erfasst und dem Bundesamt für Sicherheit im Gesundheitswesen in Wien (Formular: “Meldung über eine Arzneimittel-Nebenwirkung“) gemeldet.

**6 Dokumentation**

**6.1 Erhebungsbogen**

Beim Erhebungsbogen handelt es sich um einen papierbezogenen Case Report Form.

**6.2 Prüfordner**

Alle für die vorliegende Studie erforderlichen Dokumente (CRF, Daten der toxikologischen Untersuchung, Genotypisierungsdaten u.a.) werden durch den klinischen Prüfer katalogisiert und nach Anonymisierung durch Kodierung in einem Prüfordner aufbewahrt. Der Schlüsselcode liegt beim Projektverantwortlichen. Es wird sichergestellt, dass die Daten der absoluten Geheimhaltung unterliegen und vor dem Zugriff Unbefugter geschützt sind.

Im Rahmen des Monitorings wird der Prüfordner auf Aktualität und Vollständigkeit entsprechend den Regularien überprüft.

**6.3 Aufbewahrung der Daten**

Die Aufbewahrung der Daten erfolgt durch den Prüfer. Die kodiert anonymisierten Daten werden 10 Jahre aufbewahrt und anschließend fachgerecht vernichtet.

**7 Monitoring**

Der Prüfer erklärt sich bereit, dass der Monitor im Abstand von 16 Wochen eine Datenüberprüfung vornimmt, um die zufrieden stellende Erhebung der Daten und die Einhaltung des Prüfplanes zu sichern.

Aufgaben des Monitors sind:

- Prüfung, ob das Prüfzentrum den Anforderungen der klinischen Prüfung entspricht (Patientenpopulation, Geräte, Aufbewahrung des Studienmaterials)
- Überprüfung des Prüfordners auf Vollständigkeit und Aktualität
- Originaldatenabgleich

**8 Dateneingabe, Datenmanagement**

Die Daten werden in einer Studienmappe dokumentiert und in weiterer Folge mit MS Exel und SPSS-Software verwaltet und (statistisch) bearbeitet.

Es wird eine manuelle/visuelle Überprüfung auf medizinische Plausibilität der Daten durchgeführt.

**9 Statistik**

**10.1 Fallzahlplanung**

Für genetische Polymorphismen im Zusammenhang mit den Dosierungsschemata der Drogenersatztherapie gibt es noch keine verwertbaren Publikationen. Eine seriöse Fallzahlschätzung kann daher zum jetzigen Zeitpunkt nicht durchgeführt werden. Genetische Polymorphismen mit einer Prävalenz von > 10 % werden mittels der unten angeführten statistischen Methoden untersucht.

**10.2 Statistische Methoden**

- Zielgrößen

Hauptzielkriterium: Dosierung (mg pro kg Körpergewicht pro Tag) der Substitutionsopioide bzw. -opiate

Nebenzielkriterium: Plasmakonzentrationen (ng/ml) der Substitutionsopioide bzw. -opiate

- Einflussgrößen

Genetische Polymorphismen in folgenden Genen: OPRM1-, OPRK1-, STAT6-, MDR1- COMT, MC1R, 5HT1A, UGT1A1-, UGT1A3-Gen.

- Störgrößen

Dauer der Opioidabhängikeit, Geschlecht (Arzt wird keine Störgröße darstellen: kein Untersuchereffekt und verblindet)

- Verwendete statistische Verfahren

Schritt 1: uni- und multivariate Verfahren der Assoziationsanalyse v.a. Mixed Effects Modelle

Schritt 2: Adjustierung für Störgrößen (Erhebung durch CRF) in multivariaten Regressionsmodellen.

**10 Berichterstattung**

**10.1 Studienbericht**

Die Studienbericht erfolgt nach Empfehlungen der ICH-GCP E6: ICH Guideline for Structure and Content of Clinical Study Reports.

**10.2. Publikationen**

Die Präsentation der Ergebnisse im Rahmen von Publikationen in wissenschaftlichen Journalen (peer reviewed) wird angestrebt und soll mindestens dem in den Consolidated Standards of Reporting Trials (CONSORT Statement) festgelegten Umfang entsprechen. Mit Einverständnis aller genannten Autoren ist auch die Publikation von Teilergebnissen vorgesehen.

**11 Ethische Aspekte**

**11.1 Votum der Ethikkommission**

Ein positives Votum der Ethikkommission der Medizinischen Universität Innsbruck ist Voraussetzung für die Durchführung der vorliegenden Studie.

**11.2 Patienteninformation und Einverständniserklärung**

Die Rekrutierung der Patienten erfolgt im Rahmen der regulären Sprechstunde der Ambulanz für Abhängigkeitserkrankungen. Vor Studieneinschluss erfolgt ein ausführliches Informationsgespräch mit detaillierter Beantwortung allfälliger Fragen. Anschließend wird die schriftliche „Patienteninformation und Einwilligungserklärung zur Teilnahme an einer klinischen Studie“ vorgelegt.

**11.3 Patientenversicherung**

Für die Patienten besteht ein Versicherungsschutz (Personenschadenversicherung nach § 32 (1), Pkt. 11 AMG) durch die Zürich Versicherungs-Aktiengesellschaft (Polizzennummer 07225462-7).

**11.4 Datenschutz und Verschwiegenheitspflicht**

Personenbezogene Daten dürfen im Rahmen der klinischen Studie nur mit ausdrücklicher Zustimmung des einzelnen Probanden verwendet werden. Die Verarbeitung erfolgt ausschließlich in anonymisierter Form.

Zugang zu den personenbezogenen Daten haben nur der Prüfer und dessen Mitarbeiter. Alle Personen, die aufgrund ihrer beruflichen Tätigkeit Zugang zu diesen Daten haben sind – unbeschadet anderer gesetzlicher Verschwiegenheitspflichten – gemäß §15 DSG 2000 an das Datengeheimnis gebunden.

**11.5 Aufbewahrung des Probenmaterials**

Wir beabsichtigen, das Blut- und DNA-Probenmaterial auch nach Ende dieses Forschungsprojekts für weitere Untersuchungen der Opioidabhängigkeitserkrankung oder Erkrankungen die mit der Opioidabhängigkeitserkrankung assoziiert sind aufzubewahren. Voraussetzung für fortlaufende Untersuchungen ist ein weiteres positives Votum der Ethikkommission. Wir möchten uns auch die Möglichkeit vorbehalten, die Proben an andere europäische Forschungszentren zu wissenschaftlichen Zwecken und ausschließlich in anonymisierter Form weiterzugeben, sofern die lokale Ethikkommission der Empfängerinstitution der Verwendung der Proben zu wissenschaftlichen Zwecken zustimmt.

Der einzelne Proband wird im Rahmen der Aufklärung gesondert auf die Aufbewahrung und internationale Weitergabe der Proben hingewiesen und kann auch gesondert diesbezüglich einwilligen oder verweigern. Sollten der einzelne Proband mit der Aufbewahrung von Probenmaterial und mit der Weitergabe des Probenmaterials an andere europäische Forschungszentren nicht einverstanden sein, werden seine Proben nach Durchführung der in diesem Projekt geplanten Untersuchungen vernichtet. Die Verantwortung für die Vernichtung des Materials übernimmt Frau Dr.med. Beate Beer.

**SUBSTUDIE 1**

**HÄUFIGKEITEN GENETISCHER POLYMORPHISMEN IN POPULATIONEN MIT UND OHNE OPIOIDABHÄNGIGKEITSER-KRANKUNG**

**2 Ziel der Substudie**

**2.1 Primäres Studienziel**

Ziel der Substudie ist, die Häufigkeit bestimmter Genpolymorphismen in den eingangs angeführten Genen (insbesondere im OPRK1-Gen, auch im OPRM1-, OPRD1-, ADRBK2-, MDR1-, STAT6-, COMT-, GAL-, MC1R-, 5HTR1A-, DRD2-, SCLO1B1-, SCLO1A2-, UGT2B7-, UGT1A1- und UGT1A3-Gen) bei Personen mit einer Opioidabhängigkeitserkrankung zu untersuchen (anhand der Genotypisierungsdaten der Hauptstudie) und diese mit der Häufigkeit in einer vergleichbaren Kontrollpopulation zu vergleichen.

Es gibt erste Hinweise, dass bestimmte Polymorphismen in Opioidrezeptor-Genen (OPRK1 36G>T, OPRM1 118A>G) bei Opioidabhängigen häufiger vorkommen bzw. mit einer erhöhten Vulnerabilität für Suchterkrankungen einhergehen. Die Allelhäufigkeiten von Polymorphismen im COMT-, MDR1- und STAT6-Gen bei Patienten mit einer Opioidsuchterkranung wurden unseres Wissens jedoch noch nicht der Allelhäufigkeit einer vergleichbaren Kontrollpopulation gegenübergestellt. Durch diese Substudie können weitere Erkenntnisse über eine allfällige Häufung bestimmter genetischer Polymorphismen bei Patienten mit Suchterkrankungen gewonnen werden.

**3.2 Beschreibung der Substudie**

**3.1 Studiendesign**

Die Substudie entspricht einer kontrollierten explorativen Studie.

**3.2 Primäre Hypothese**

Der OPRK1 36G>T Polymorphismus ist häufiger bei opioidabhänigen Patienten vorhanden als bei einer Kontrollpopulation.

**3.3. Sekundäre Hypothese**

Polymorphismen im OPRM1-, OPRD1-, ADRBK2-, MDR1-, STAT6-, COMT-, GAL-, MC1R-, 5HTR1A-, DRD2-, SCLO1B1-, SCLO1A2-, UGT2B7-, UGT1A1- und UGT1A3-Gen kommen häufiger bei opioidabhängigen Patienten vor als bei einer vergleichbaren Kontrollpopulation.

**3.4 Patientenzahlen**

Es werden die Genotypisierungsdaten der Patienten der Hauptstudie in die Studie eingeschlossen. Zur Fallzahlschätzung s.9.1.

**3.5 Kontrollpopulation**

Die Teilnehmer werden bezüglich der Alters-, Geschlechts- und ethnischen Verteilung an das Patientenkollektiv angepasst. Es werden ca. 200 Teilnehmer in die Studie eingeschlossen.

**3.6 Zeitplan**

- Patientenrekrutierung: siehe Hauptstudie
- Rekrutierung der Kontrollen: 1,5 Jahre
- Follow-Up Phase 1 Jahr
- Vorgesehener Zeitpunkt des Studienbeginns: WS 2008
- Voraussichtlicher Zeitpunkt des Studienendes: WS 2012

Über- oder Unterschreitungen des Zeitplans werden keinen Einfluss auf die Studie nehmen.

**4 Studienpopulation**

- 1. **Einschlusskriterien Patienten**

Siehe Hauptstudie.

- 1. **Ausschlusskriterien Patienten**

Siehe Hauptstudie.

- 1. **Einschlusskriterien Kontrollgruppe**
- keine Abhängigkeitserkrankung (Alkoholabhängigkeit)
- kein Missbrauch illegaler Substanzen in der Vorgeschichte
- Alter >18 Jahre, < 50 Jahre
- Geschäftsfähigkeit bei Studieneinschluss
- unterzeichnete Einverständniserklärung
- inkludiert werden Männer und Frauen

**4.4 Ausschlusskriteren Kontrollgruppe**

- Abhängigkeitserkrankung (Alkoholabhängigkeit)
- Missbrauch illegaler Substanzen in der Vorgeschichte
- fehlende Geschäftsfähigkeit bei Studieneinschluss

**5 Ablauf der Substudie**

**5.1 Patienten**

Die durch die Hauptstudie erhaltenen Genotypisierungsdaten werden für diesen Teil der Studie übernommen.

**5.2 Kontrollgruppe**

Die Rekrutierung der Teilnehmer erfolgt über den Aufruf zur freiwilligen Teilnahme (z.B. Mitarbeiter, Studenten). Vor Studieneinschluss erfolgt ein ausführliches Informationsgespräch mit detaillierter Beantwortung allfälliger Fragen. Anschließend wird eine schriftliche „Probandeninformation und Einwilligungserklärung zur Teilnahme an einer klinischen Studie“ vorgelegt.

Bei Einwilligung zur Teilnahme und dem Vorliegen einer unterzeichneten Einverständniserklärung werden für die genetischen Untersuchungen mittels Filzkämmen zwei Mundhöhlenabstriche (MHA) zur Gewinnung der DNA abgenommen. Dies erfolgt einmalig und ist völlig schmerzlos und risikofrei.

Die Probanden können jederzeit ohne Angabe von Gründen aus der Studie ausscheiden.

**Zeitplan für die Probanden der Kontrollgruppe:**

| **KONTROLLPOPULATION** | Woche 1 | Monat 1-6 | Monat 7-18 |
| --- | --- | --- | --- |
| Informationsgespräch | x |  |  |
| Mundhöhlenabstrich | x |  |  |
| Labor |  | x |  |
| Auswertung |  |  | x |

**6 Dokumentation**

Siehe Hauptstudie.

**7 Monitoring**

Siehe Hauptstudie.

**8 Dateneingabe, Datenmanagement**

Siehe Hauptstudie.

**9 Statistik**

**9.1 Fallzahlplanung**

Die Abschätzung der Fallzahl erfolgt auf der Basis einer Studie von Gerra et al.Error: Reference source not found (Allelhäufigkeit des OPRK1 36G>T Polymorphismus von 10,4% bei opioidabhängigen Personen versus 4,3% bei der Kontrollpopulation). Um diesen Unterschied als statistisch signifikant erkennen zu können ist ein Patientenkollektiv von n=200 pro Gruppe (Patienten und Kontrollen) erforderlich. Bei einem geschätzten Drop-out von ca. 10% werden daher primär 200 Teilnehmer pro Gruppe in die Studie aufgenommen.

**9.2 Statistische Methoden**

- Zielgrößen

Hauptzielkriterium: Allelhäufigkeit des OPRK1 36G>T Polymorphismus

Nebenzielkriterien: Allelhäufigkeit genetischer Polymorphismen in den Genen OPRM1, OPRD1, ADRBK2, MDR1, STAT6, COMT, GAL, MC1R, 5HTR1A, DRD2, SCLO1B1, SCLO1A2, UGT2B7, UGT1A1 und UGT1A3.

- Verwendete statistische Verfahren

Chi-Quadrat Test, Kontingenztafelanalyse. Logistische Regression

**10 Berichterstattung**

Siehe Hauptstudie.

**11 Ethische Aspekte**

Siehe Hauptstudie.

**SUBSTUDIE 2**

**UNTERSUCHUNG PHARMAKOGENETISCHER ASPEKTE BEI OPIAT- bzw. OPIOIDINDUZIERTEN DIREKTEN DROGENTODESFÄLLEN**

**2. Ziel der Substudie 2**

**2.1. Primäres Studienziel**

Das primäre Ziel der Studie ist es, an einem Kollektiv von opiatbezogenen Todesfällen zu untersuchen, ob und welche pharmakogenetischen Polymorphismen das individuelle Risiko einer opiatbedingten zentralen Atemlähmung beeinflussen können. Zu diesem Zweck sollen Polymorphismen in **15** **pharmakologisch relevanten Genen** (OPRM, OPRD, OPRK, COMT, STAT6, MDR1, UGT1A1, DRD2, GAL, SLCO1B1, SLCO1A2, MC1R, UGT2B7, 5-HTR1A, GRK3) untersucht werden (s. a. Kap. 1).

**2.2 Sekundäre Studienziele**

Zusätzlich sollen im Rahmen dieser Studie weitere Aspekte des Drogentodes untersucht werden und damit folgende sekundäre Ziele erreicht werden:

- Korrelation der post-mortem Konzentrationsverhältnisse von Opiaten/Opioiden in Blut und Liquor mit Polymorphismen in den Transportproteingenen MDR1, SLCO1B1 und SLCO1A2
- Untersuchung von Muttersubstanz/Metaboliten-Ratios in Abhängigkeit von Polymorphismen in Genen, die für metabolisierende Enzyme (UGT2B7, UGT1A1) kodieren
- tieferes Verständnis von potentiell letalen Substanzblutspiegeln und insbesondere auch potentiell tödlichen Substanzkombinationen
- Erfassung von eventuell vorhandenen geschlechtsspezifische Unterschieden insbesondere bezüglich Blutspiegel und Substanzkombinationen

**3.2 Beschreibung der Substudie**

**3.1 Studiendesign**

Die Substudie entspricht einer unkontrollierten explorativen bizentrischen Studie.

**3.2 Primäre Hypothese**

Bestimmte Polymorphismen in den Genen OPRM, OPRD, OPRK, COMT, STAT6, MDR1, UGT1A1, DRD2, GAL, SLCO1B1, SLCO1A2, MC1R, UGT2B7, 5-HTR1A und GRK3 kommen bei Drogentoten mit niedrigen Opioidkonzentrationen in den post-mortem Asservaten häufiger/seltener vor als bei Drogentoten mit hohen Opioidkonzentrationen in den post-mortem Asservaten.

**3.3 Sekundäre Hypothesen**

- Das Vorliegen bestimmter genetischer Polymorphismen im MDR1/SLCO1B1/SLCO1A2 Gen beeinflusst das Konzentrationsverhältnis von Methadon zwischen peripherem Blut und Liquor.
- Das Vorkommen bestimmter genetischer Polymorphismen im MDR1/SLCO1B1/SLCO1A2 Gen beeinflusst das Konzentrationsverhältnis von Heroin/Morphin bzw. deren Metaboliten (Monoacetylmorphin, Morphin, Morphin-3-Glucuronid, Morphin-6-Glucuronid) zwischen peripherem Blut und Liquor.
- Das Vorliegen bestimmter genetischer Polymorphismen im UGT2B7/UGT1A1 Gen beeinflusst das Verhältnis von Morphin zu Morphin-3- bzw. -6-glucuronid im Blut

**3.4 Obduktionsfälle bzw. –asservate (Stichprobe)**

Bei der geplanten Studie setzt sich das Studienkollektiv aus direkten Drogentodesfällen zusammen, bei denen Methadon, Heroin oder Morphin als Hauptsubstanz identifiziert wurde. Von den routinemäßig aufbewahrten fallspezifischen Obduktionsasservaten sollen ca. 1 ml peripheres Blut, 1 ml Liquor und 10 ml Harn für die studienbezogenen toxikologischen Analysen verwendet werden. Zudem sollen ca. 1 ml peripheres Blut für die studienbezogenen genetischen Analysen verwendet werden. Voraussichtlich werden ca. 300 opiat-/opioid-assoziierte Drogentodesfälle in die Studie eingeschlossen. Diese Fälle sollen am Institut für Rechtsmedizin in Hamburg sowie dem Institut für Gerichtliche Medizin in Innsbruck über einen Zeitraum von etwa 24 Monaten gesammelt und in weiterer Folge bezüglich der genannten wissenschaftlichen Fragestellungen untersucht werden.

**3.5 Wichtiger Hinweis**

Für die Substudie 2 sind **keine zusätzlichen bzw. studienspezifischen Maßnahmen bei der routinemäßig durchgeführten Obduktion erforderlich**. Die für die genetischen und toxikologischen Zusatzuntersuchungen herangezogenen Obduktionsproben werden von den im Rahmen der gerichtsmedizinischen Routine aufbewahrten Obduktionsasservaten entnommen.

**3.6 Zeitplan**

- Sammeln der Drogentodesfälle: 2 Jahre
- Untersuchungsphase: 1 Jahr (beginnt während der Sammelphase)
- Follow-Up Phase 1 Jahr
- Vorgesehener Zeitpunkt des Studienbeginns: Jänner 2010
- Voraussichtlicher Zeitpunkt des Studienendes: Juli 2013

Über- oder Unterschreitungen des Zeitplans werden keinen Einfluss auf die Studie nehmen.

**4. Studienpopulation**

Bei suchtgiftbezogenen Todesfällen sind insbesondere Heroin, Morphin und Methadon als die quantitativ bedeutsamsten Substanzen zu betrachten. Aufgrund der pharmakologischen Ähnlichkeit von Heroin und Morphin (Morphin ist der Hauptmetabolit von Heroin), deren pharmakokinetischen Eigenschaften sich wiederum deutlich von jenen des Methadons abgrenzen, sollen die Drogentodesfälle in zwei verschiedene Studienkollektive aufgeteilt werden: Heroin/Morphin - und Methadon-assoziierte Todesfälle.

**4.1 Einschlusskriterien Methadongruppe**

- Vorliegen eines direkten Drogentodes
- Methadon als toxikologische Hauptsubstanz in den Obduktionsasservaten
- eventuell zusätzlich vorhandene Substanzen im peripheren Blut in einem nicht-toxischen Konzentrationsbereich
- Lebensalter < 50 Jahre

**4.2 Einschlusskriterien Heroin-/Morphingruppe**

- Vorliegen eines direkten Drogentodes
- Heroin/Morphin als toxikologische Hauptsubstanz in den Obduktionsasservaten
- eventuell zusätzlich vorhandene Substanzen im peripheren Blut in einem nicht-toxischen Konzentrationsbereich
- Lebensalter < 50 Jahre

**4.3 Ausschlusskriterien Methadon- und Heriongruppe**

- indirekter Drogentod
- zusätzliche Substanzen im peripheren Blut in toxischen Konzentrationen

**5 Untersuchungsumfang**

Für die geplante Studie ist keine Entnahme von zusätzlichen Leichensasservaten erforderlich. Das für die geplanten Untersuchungen benötigte biologische Material (Blut, Liquor, Harn) wird routinemäßig im Rahmen der gerichtsmedizinischen Obduktion asserviert und für einen Zeitraum von 2 Jahren aufbewahrt. Für die Studie wird nur ein kleiner Anteil (2 ml Blut, 1 ml Liquor, 10 ml Harn) der Gesamtmenge der Obduktionsasservate verwendet.

Die studienspezifischen Laboruntersuchungen werden am Institut für Gerichtliche Medizin in Innsbruck durchgeführt.

**5.1 Toxikologische Untersuchungen**

Im Rahmen der studienbezogenen toxikologischen Untersuchungen wird die post-mortem Konzentration von Methadon, Morphin bzw. Heroin und deren Metaboliten in Blut, Liquor und Harn quantitativ erfasst. Zudem ist geplant, eventuelle zusätzliche Substanzen (z.B. Benzodiazepine, andere Suchtmittel), die einen wesentlichen Beitrag zum tödlichen Ausgang geleistet haben können, quantitativ zu erfassen.

Die toxikologischen Bestimmungen erfolgen nach Festphasenextraktion mit geeigneten analytischen Methoden (Immunoassay, Flüssigkeitschromatographie-Massenspektrometrie, Gaschromato-graphie-Massenspektrometrie).

**5.2 Pharmakogenetische Untersuchungen**

Für die geplanten molekularbiologischen Untersuchungen in den genannten pharmakologisch relevanten Genen soll jeweils die DNA aus 1 ml Blut extrahiert werden. Anschließend sollen die spezifischen Genabschnitte mittels PCR amplifiziert werden. Die PCR Amplifikate werden dann mittels einer auf Massenspektrometrie basierenden Methode (ICEMS) typisiert. Es werden nur Polymorphismen in den genannten Genen typisiert. Es werden keine zusätzlichen genetischen Informationen gewonnen.

**6 Dokumentation**

siehe Hauptstudie. Bei dem papierbezogenen Case report form handelt es sich hauptsächlich um eine komprimierte Form des sog. Formulars der Österreichischen Gesellschaft für Gerichtsmedizin, das der bundesweiten zentralen Erfassung von Drogentodesfällen durch das Österreichische Bundesinstitut für Gesundheitswesen (ÖBIG) dient.

**7 Dateneingabe, Datenmanagement**

siehe Hauptstudie

**8 Statistik**

**8.1 Fallzahlplanung**

Für pharmakogenetische Polymorphismen im Zusammenhang mit opiat-/opioidbezogenen Todesfällen gibt es noch keine verwertbaren Daten. Eine seriöse Fallzahlschätzung kann daher zum jetzigen Zeitpunkt nicht durchgeführt werden. Genetische Polymorphismen mit einer Prävalenz von > 5 % werden mittels der unten angeführten statistischen Methoden untersucht.

**8.2 Statistische Methoden**

- Zielgrößen

Hauptzielkriterium: post-mortem Konzentration von Methadon/Heroin und deren Metaboliten in peripherem Blut

Nebenzielkriterium: post-mortem Konzentration von Methadon/Heroin und deren Metaboliten in Liquor/Harn

- Einflussgrößen

Genetische Polymorphismen in folgenden Genen: OPRM, OPRD, OPRK, COMT, STAT6, MDR1, UGT1A1, DRD2, GAL, SLCO1B1, SLCO1A2, MC1R, UGT2B7, 5-HTR1A und GRK3

- Störgrößen

Zweitsubstanz(en) in post-mortem Blut bzw. Liquor, Geschlecht

- Verwendete statistische Verfahren

Schritt 1: uni- und multivariate Verfahren der Assoziationsanalyse v.a. Mixed Effects Modelle

Schritt 2: Adjustierung für Störgrößen in multivariaten Regressionsmodellen.

**9 Berichterstattung**

siehe Hauptstudie

**10 Ethische Aspekte**

**10.1 Votum der Ethikkommission**

Ein positives Votum der jeweiligen lokalen Ethikkommission (Medizinischen Universität Innsbruck, Universitätsklinikum Hamburg-Eppendorf*)* ist Voraussetzung für die Durchführung der vorliegenden Studie.

**10.2 Datenschutz und Verschwiegenheitspflicht**

Die Behandlung der Drogentodesfälle im Rahmen der Studie erfolgt **ausschließlich in anonymisierter Form**. Die zu untersuchenden Leichenasservate werden umgehend durch Kodierung anonymisiert. Für die Auswertung der Studienergebnisse werden das Alter, das Geschlecht sowie erhobene Obduktionsbefunde herangezogen. Allfällig zusätzlich bekannte persönliche Daten zur Leiche dürfen im Rahmen der Studie nicht verwendet werden.

Zugang zu den studienbezogenen Daten haben nur der Prüfer und dessen Mitarbeiter. Alle Personen, die aufgrund ihrer beruflichen Tätigkeit Zugang zu diesen Daten haben sind – unbeschadet anderer gesetzlicher Verschwiegenheitspflichten – gemäß §15 DSG 2000 an das Datengeheimnis gebunden.

Es werden keine studienspezifischen Daten an Angehörige der Drogentoten weitergegeben.

**10.3 Aufbewahrung des Probenmaterials**

Die fallspezifischen Obduktionsasservate werden im Rahmen der gerichtsmedizinischen Routine bzw. aus rechtlichen Gründen (eventuell notwendige Zusatzuntersuchungen) für 2 Jahre aufbewahrt. Wir möchten uns die Möglichkeit vorbehalten, die Obduktionsasservate auch nach Ende dieser Frist für eventuelle weitere wissenschaftliche Untersuchungen bezüglich der toxikologischen Aspekte des Drogentodes aufzubewahren. Voraussetzung für fortlaufende Untersuchungen ist ein weiteres positives Votum der Ethikkommission.

1. Wolf CR, Smith F, Smith RL: Science, medicine, and the future: pharmacogenetics. *BMJ* 320: 987-990, 2000. [↑](#footnote-ref-2)
2. Smith HS: Variations in opioid responsiveness. *Pain Physician* 11: 237-248, 2008. [↑](#footnote-ref-3)
3. Mattick RP, Breen C, Kimber J, et al. Methadone maintenance therapy versus no opioid replacement therapy for opioid dependence. *Cochrane Database of Systematic Reviews* 2: CD002209, 2003. [↑](#footnote-ref-4)
4. Krantz JM, Mehler PS. Treating opioid dependence. growing implications for primary care. *Archives of Internal Medicine* 164: 277-288, 2004. [↑](#footnote-ref-5)
5. Haas S, Busch M, Horvath I, Türscherl E, Weigl M, Wirl C: Bericht zur Drogensituation 2007. *Gesundheit Österreich GmbH, Geschäftsbereich ÖBIG*, 2007. [↑](#footnote-ref-6)
6. Doran CM, Shanahan M, Mattick RP, Ali R, White J et al. Buprenorphine versus methadone maintenance: a cost-effectiveness analysis. *Drug Alcohol Dependence* 71: 292-302, 2003. [↑](#footnote-ref-7)
7. Wolff K. Characterization of methadone overdose: clinical considerations and the scientific evidence. Therapeutic Drug Monitoring 24: 457-470, 2002. [↑](#footnote-ref-8)
8. Soyka M, Zingg C, Koller G, Kuefner H. Retention rate and substance use in methadone and buprenorphine maintainance tharapy and predictors of outcome: results from a randomized study. *International Journal of Neuropsychopharmacology* 21:1-13, 2008. [↑](#footnote-ref-9)
9. Connock M, Juarez-Garcia A, Jowett s, Frw E, Lio Z, Taylor RJ, Fry-Smith A, Day E: Methadone and buprenorphine for the management of opioid dependence: a systematic review and economic evaluation. *Health Technology Assessment* 11, 2007. [↑](#footnote-ref-10)
10. Somogyi A, Barratt D, Coller JK: Pharmacogenetics of Opioids. *Clinical Pharmacology & Therapeutics* 81: 429-444, 2007. [↑](#footnote-ref-11)
11. Campa D, Gioia A, Tomei A, Poli P Barale R: Association of ABCB1/MDR1 and OPRM1 Gene Polymorphisms with Morphine Pain Relief. *Clinical Pharmacology & Therapeutics* 83: 559-566, 2008. [↑](#footnote-ref-12)
12. Coller JK, Barratt DT, Dahlen K, Loennechen MH, Somogyi AA. ABCB1 genetic variability and methadone dosage requirements in opioid-dependent individuals. Clinical Pharmacology & Therapeutics 80:682-690. 2006. [↑](#footnote-ref-13)
13. Wen-Ying C, Cheng-Haung W, Ping-Hsin L, Chien-Cheng L et al. Human Opioid Receptor A118G Polymorphism Affects Intravenous Patient-controlled Analgesia Morphine Consumption after Total Abdominal Hysterectomy. *Anestesiology* 105:334-337: 2006. [↑](#footnote-ref-14)
14. Klepstad P, Rakvag T, Kaasa S, Holthe M, Dale O et al. The 118A>G polymorphism in the human µ-opioid receptor gene may increase morphine reyuirements in patients with pain caused by malignangt disease. Acta Anaesthesiologica Scandinavica 48: 1232-1239, 2004. [↑](#footnote-ref-15)
15. Gerra F, Leonardi C, Cortese E, D’Amore A, Lucchini A, Strepparola F et al. Human Kappa Opioid Receptor Gene (OPRK1) Polymorphism Is Associated With Opiate Addicition. American Journal of Medical Genetics Part B (Neuropsychiatric Fenetics) 144B:771-775: 2007. [↑](#footnote-ref-16)
16. Yuferov V, Fussell D, LaForge S, Niesen DA, Gordon D et al. Redefinition of the human kappa opioid receptor gene (OPRK1) structure and association of haplotypes with opiate addiction. *Pharmacogenetics* 14:73-804:2004. [↑](#footnote-ref-17)
17. Ross JR, Rutter D, Welsh K, Joel SP, Goller K, Wells AU, Du Bois R, Riley J: Clinical response to morphine in cancer patients and genetic variation in candidate genes. *The Pharmacogenomics Journal* 5:324-336, 2005. [↑](#footnote-ref-18)
18. OMIM 116790 [↑](#footnote-ref-19)
19. Rakvag T, Klepstad P, Baar C, Kvam T, Dale O, Kaasa S et al. The Val158Met polymorphism of the human catechol-O-methylransferase (COMT) gene may influence morphine requirements in cancer pain patients. Pain 116:73-78, 2005. [↑](#footnote-ref-20)
20. Coller JK, Barratt DT, Dahlen K, Loenechen MH, Somogyi A. ABCB1 genetic variability and methadone dosage requirements in opioid-dependent individuals. Journal of Clinical Pharmacology and Therapeutics 80:682-6890, 2006. [↑](#footnote-ref-21)
21. Jada SR, Lim R, Wong CI, Shu X, Lee SC, Zhou Q, Goh BC, Chowbay B: Role of UGT1A1*, UGT1A1*28 and ABCG2c.421C>A polymorphisms in irinotecan-induced neutropenia in Asian cancer patients. *Cancer Science* 98:1461-1467, 2007. [↑](#footnote-ref-22)
22. Iwai M, Maruo Y, Ito M, Yamamoto K, Sato H, Takeuchi Y. Six novel UDP-glucuronosyltransferase (UGT1A3) polymorphisms with varying activity. *Journal of Human Genetics 49*: 123-128: 2004. [↑](#footnote-ref-23)
23. Mogil JS, Ritchie J, Smith SB: Melanocortin-1 receptor gene variants affect pain and µ-opioid analgesia in mice and humans. *Journal of Medical Genetics* 42: 583-587, 2005. [↑](#footnote-ref-24)
24. Drago A, Ronchi DD, Serretti A: 5-HT1A gene variants and psychiatric disorders: a review of current literature and selection of SNPs for future studies. *International Journal of Neuropsychopharmacology* 11: 701-721, 2008. [↑](#footnote-ref-25)
25. Kreek MJ, Bart F, Lilly C, Laforge S, Nielsen D. Pharmacogenetics and Molecular Genetics of Opiate and Cocaine Addictions and Their Treatments. *Pharmacological Reviews* 57: 1-26, 2005. [↑](#footnote-ref-26)
26. Xu K, Nagarjann S, Gu X, Goldman D: Relationship of the Delta-Opioid Receptor Gene to Heroin Abuse in a Large Chinese Case/Control Sample. *American Journal of Medical Genetics* 110: 45-40, 2002. [↑](#footnote-ref-27)
27. Gesondheit Österreich GmbH GÖG: Suchtgiftzezogene Todesfälle, 2008. [↑](#footnote-ref-28)
28. Europäische Beobachtungsstelle für Drogen und Drogensucht Jahresbericht 2008: Stand der Drogenproblematik in Europa, 2008. [↑](#footnote-ref-29)
29. Kjelsberg E, Winther M, Dahl AA. Overdose deaths in young substance abusers: accidents or hidden suicides? Acta Psychiatr Scand: 236-42, 1995. [↑](#footnote-ref-30)
30. Farrell M, Neeleman J, Griffiths P, Strang J. Suicide and overdose among opiate addicts. Addiction: 321-323, 1996. [↑](#footnote-ref-31)
31. Bayrische Akademie für Suchtfragen. Prävention von Drogentodesfällen: Fakten, Zahlen und Beispiele aus der Praxis, 2009. [↑](#footnote-ref-32)
32. Shields L, Hunsaker J, Corey T, Stewart D. Methadone Toxicity Fatalities: A Review of Medical Examiner Cases in a Large Metropolitan Area. Journal of Forensic Sciences, 52, 2007. [↑](#footnote-ref-33)
33. Caplehorn J, Drummer O. Methadone dose and post-mortem blood concentration. Drug and Alcohol Review 21: 329-333, 2002. [↑](#footnote-ref-34)
34. Jagsch R, Gombas W, Schindler S, Eder H et al. Opioid plasma concentrations in methadone- and buprenorphine-maintained patients. Addiction Biology 10: 365-371, 2005. [↑](#footnote-ref-35)
35. Vidal Disc Österreich, Oktober 2007. [↑](#footnote-ref-36)
