# Supplementary material for: Association of Polymorphisms in Pharmacogenetic Candidate Genes (OPRD1, GAL, ABCB1, OPRM1) with Opioid Dependence in European Population: A Case-Control Study
Source: PLoS One. 2013 Sep 25;8(9):e75359. doi: 10.1371/journal.pone.0075359 (PMC3783401; doi:10.1371/journal.pone.0075359)
Supplement: Supporting Information S1 — (DOC) [file pone.0075359.s002.doc]

**A novel amplification strategy for genotyping with liquid chromatography-electrospray ionization mass spectrometry**

Beate Beer^1,+^, Kaarel Krjutškov^2,+^, Robert Erb^1^, Triin Viltrop^3^, Herbert Oberacher^1,*^

^1^ Institute of Legal Medicine, Innsbruck Medical University, Innsbruck 6020, Austria

^2^ Estonian Genome Center, University of Tartu, Tartu 50410, Estonia

^3^ Clinic of Hematology and Oncology, University of Tartu, Puusepa 6, Tartu 51014, Estonia

^+^ Beate Beer and Kaarel Krjutškov contributed equally to this work.

* Corresponding author:

Assoc.Prof. Dr. Herbert Oberacher

Institute of Legal Medicine, Innsbruck Medical University, Muellerstrasse 44, 6020 Innsbruck, Austria.

E-mail: herbert.oberacher@i-med.ac.at

Tel.: +43 (0)512 9003 70639

Fax: +43 (0)512 9003 73600

.

**Keywords:** mass spectrometry, liquid chromatography, polymerase chain reaction, pharmacogenetics, SNP genotyping

**Abstract**

Among numerous available genotyping techniques, mass spectrometry (MS) based methods play a major role in providing high quality genotype data at reasonable costs for research and diagnostics, e.g. for pharmacogenetic applications. Ion-pair reversed-phase liquid chromatography hyphenated to electrospray ionization time-of-flight MS (ICEMS) is, for example, a powerful instrument allowing for a direct characterization of complex mixtures of polymerase chain reaction (PCR) amplified DNA fragments. Current limitations of PCR-ICEMS genotyping mainly concern multiplex PCR set-up. Assay development often requires time-consuming primer design and intensive optimization of PCR conditions. To overcome this restraint, a robust amplification strategy originally combined with arrayed primer extension genotyping was transferred and adapted to ICEMS genotyping. The modifications involved limitation of the primer length, application of two universal sequences and amplification with a proof-reading DNA polymerase. To demonstrate the applicability of the novel amplification strategy for ICEMS, a 23-plex pharmacogenetic genotyping assay was developed. After slight optimization steps an efficient and quantitatively balanced amplification of all targeted markers was achieved, resulting in a convenient characterization of the multiplexed PCR fragments with ICEMS. Expenditure of time, costs and hands-on work associated with assay design and optimization was dramatically lowered compared to previous multiplex PCR-ICEMS assays. The developed 23-plex assay was applied in a pharmacogenetic study including 284 individuals (genotype call rate 99.0%). A total of 399 SNPs were retyped by Sanger sequencing (concordance rate 99.8%). The PCR-ICEMS assay turned out to be an accurate, reliable, cost-effective and a ready-to-use tool for pharmacogenetic genotyping.

**Introduction**

With increasing knowledge of genetic variants influencing the individual drug response, genotyping of polymorphisms for pharmacogenetic studies becomes progressively more popular. At the same time, there is a strong need for analytical platforms allowing for an efficient, accurate and low-cost genotyping of pharmacogenetic variants, e.g. single nucleotide polymorphisms (SNPs).^1,2^ Efficient SNP genotyping platforms may not only provide substantial insight into the genetic determinants of aberrant drug responses, but could also serve as important tools for the realization of the personalized medicine approach.^3-5^ Among the numerous genotyping techniques that have been established so far, mass spectrometry (MS) based methods play a major role in providing high quality genotype data at reasonable costs.^6-13^ Beside matrix-assisted laser desorption/ionization (MALDI)-MS,^14-17^ also electrospray ionization (ESI)-MS turned out to be a powerful genotyping instrument with several attractive features.^7,18-20^ ESI-MS enables the direct genotyping of polymerase chain reaction (PCR) amplified DNA fragments based on accurate molecular mass measurements. An eminent example for ESI-MS genotyping is Ion-pair reversed-phase liquid Chromatography hyphenated to Electrospray ionization time-of-flight Mass Spectrometry (ICEMS) that enables genotyping of SNPs,^21-23^ small haplotypes,^24-27^ and short tandem repeats.^28,29^

ICEMS integrates sample preparation and mass spectrometric analysis in one method. Thus, PCR products can be purified, denatured, fractionated and accurately mass analyzed within a single run. Thereby, single nucleotide- as well as sequence length polymorphisms can easily be identified in PCR products up to 250 base pairs (bp).^30^ Furthermore, this methodology is particularly useful to characterize complex mixtures of co-amplified DNA fragments generated by multiplex PCR approaches.^26,27,29^ The combination of a multiplex PCR with ICEMS detection systems is not only helpful to increase the genotyping throughput, but also markedly reduces hands-on work and saves reagent costs. However, since the raw co-amplified PCR products are directly characterized without any post-PCR modifications, a balanced, specific and efficient amplification of all studied regions is a key factor for successful and convenient ICEMS genotyping. Thus, with increasing multiplex level, extensive assay design and optimization efforts are necessary. Primer design is often a challenging task requiring experience and careful considerations for primer kinetics, primer-primer interactions and melting temperatures (T_m_). To ensure unequivocal genotyping by molecular mass, particular attention must be paid on amplicon size (≤250 bp) and the presence of gradual mass differences between the amplicons.^23,30^ The subsequent process of assay optimization often involves multiple adjustments of primer concentrations, the reaction mixture composition and thermal cycling conditions. Sometimes, primer pairs need to be redesigned or initially intended markers need to be omitted during the optimization procedure. While a multiplex PCR represents a valuable approach to increase the genotyping throughput and decrease costs per genotype, a considerable amount of time and effort needs to be put into prior assay development. Consequently, improvements and simplification at the PCR-level of ICEMS genotyping are still eligible.

The above mentioned limitations are not specific for PCR-ICEMS assay development only and also apply to other PCR-based genotyping methods.^31-36^ In this context, many different approaches to enhance PCR multiplex capacity and to facilitate the process of assay optimization have been employed. A creative strategy is the use of hybrid primers consisting of a target-specific sequence and a non-specific universal sequence at the 5’ end.^37^ One useful property of such non-specific universal sequences is a balanced and reasonably high GC content to increase T_m_, thereby reducing primer-dimer formation and leading to a more sensitive and robust PCR.^34^ Hybrid primers enable the incorporation of the universal sequence into the sequence of the amplicon (step-1 PCR), which serves then as priming site for a complementary universal primer in a subsequent step-2 PCR (Figure 1a). By using this two-step amplification strategy, a robust 640-plex amplification assay combined with arrayed primer extension (APEX-2) as detection platform was presented recently.^35^ APEX-2 is a microarray-based genotyping approach employing a highly efficient universal sequence, a high-level multiplex thermal cycling protocol and limited amplicon sizes, thereby enabling a high-level specific primer multiplex PCR. Current limitations of the APEX-2 approach are the need for post-PCR purification and a subsequent single-base extension (SBE) reaction step, requiring additional protocol steps and expensive single-use microarrays. However, the combination of the APEX-2 PCR advantages with ICEMS genotyping platform may result in a promising, simple and cost-effective genotyping tool for research and clinical use.

In the present study, we demonstrate how the robust APEX-2 amplification strategy was transferred to the PCR-ICEMS genotyping system. Therefore, particular adaptations of the assay design and amplification strategy to mass spectrometric genotyping were made. To test the performance and reliability of the developed strategy, a 23-plex PCR assay was developed and applied to genotype 284 individuals participating in a pharmacogenetic study.

**Experimental**

**Chemicals and reagents**

Acetonitrile (HPLC gradient grade) was obtained from Sigma-Aldrich (St. Louis, MO, USA). A 1.0 M stock solution of cyclohexyldimethylammonium acetate (CycHDMMA) was prepared by titration of cyclohexyldimethylamine (Fluka, Buchs Switzerland) with acetic acid (Fluka) at 5°C until pH 8.4 was reached. For preparation of all solutions, HPLC grade water (Sigma-Aldrich) was used. All oligonucleotides were purchased from Metabion International AG (Martinsried, Germany).

**Study subjects, DNA samples and extraction**

Prior to the recruitment of participants, the study was approved by the local ethics committee (Innsbruck Medical University). All study participants provided a written informed consent. In total 142 patients undergoing opioid replacement therapy and 142 healthy individuals were recruited. DNA was extracted from collected saliva swabs using a standard Chelex extraction protocol.^38^ The DNA content of 120 samples was determined by spectrophotometry (ND-1000, Nano-Drop, Wilmington, USA) and values ranging between 15-121 ng/µl were determined.

**Development and optimization of the two-step PCR strategy dedicated to ICEMS analysis**

*(a) APEX-2 PCR amplification strategy combined with ICEMS*

The APEX-2 assay design and amplification principle is demonstrated in Figure 1a. SNP-specific primers were designed by retrieval of 50-bp genomic regions flanking either side of the SNPs of interest. A 50-bp reverse complement of the 3’→5’ strand was generated, and truncated at the 5’ end until an optimal melting temperature (57-62°C) was achieved. APEX-2 primers were generated upon addition of a universal sequence (5’-GATCAGGCGTCTGTCGTGCTC-3’) to the 5’ end of each SNP-specific primer.

The applicability of the APEX-2 amplification strategy to ICEMS genotyping was evaluated in preliminary experiments. Therefore, 5-, 10-, 15-, 20-, 25- and 30-plex PCRs using two different DNA samples as template where generated according to the two-step amplification protocol of the previously published APEX-2 640-plex genotyping assay^35^ and directly analyzed by ICEMS. The total length of the amplicons ranged between 79 and 126 bp (Table S1, Electronic Supplementary Material).

*(b) ICEMS-specific amplification strategy*

To improve its performance, the APEX-2 assay design and amplification strategy was modified and adapted to the specific requirements of ICEMS genotyping (Figure 1b). The applicability of the developed two-step PCR approach was exemplified by a 23-plex PCR assay developed for a clinical study investigating the pharmacogenetic aspects of opioid replacement therapy. The selected loci are listed in Table 1.

A key prerequisite for unequivocal mass spectrometric genotyping is the use of SNP-specific amplicon masses. Specific masses can easily be obtained by designing staggered amplicon lengths. Primer design started with the retrieval of 50-bp genomic regions flanking either side of the targeted SNPs. A major modification in comparison to the APEX-2 primer design strategy was that the length of primers was limited to 30 nts to assure successful and more balanced amplification of multiple sequences in a single PCR (step-1 PCR). Thus, for each SNP 30-bp reverse complements within the 50-bp flanking regions were generated which were truncated and shifted until inter-amplicon length intervals of at least 1 bp were achieved (Table 2). The nucleotide compositions of the obtained amplicons were used to calculate SNP-specific amplicon masses (forward and reverse strand). Due to the specific properties of the target sequence, a mass overlap of the rs7439366 amplicon with other amplicons was inevitable. This problem was solved by inserting two nucleotides (non-template addition) between the 5’ end of the specific sequence and the 3’ end of the universal sequence.

Specifity of the specific primer sequences was verified by applying the GenomeTester 1.3.^39^ The primer sequences were further assessed for the presence of SNPs using the SNPmasker software.^40^ To minimize inter-amplicon interactions during PCR amplification, two different 18 bp universal sequences were designed and added to the specific primer sequences: universal sequence 1 (5’-TACGACTCACTTAGGGAG-3’) was added to the 5’-end of each specific forward primer and universal sequence 2 (5’-CGATGTAGGTGACACTAG-3’) was added to the 5’-end of each specific reverse primer. The total length of the hybrid primers ranged between 36-46 bp. The sequences of the primers selected for the final multiplex assay are summarized in Table 2. In the step-2 PCR, the universal primer 1 (5’-CGATGTAGGTGACACTAG-3’) and 2 (5’-TACGACTCACTTAGGGAG-3’), targeting the universal sequence 1 and 2, respectively, were employed.

The specific primer amplification (step-1 PCR) involved the basic amplification of the 23 target regions and the incorporation of the universal sequence in the amplicon sequence using the specifically designed hybrid primers. The final primer concentrations in the reaction mixture are summarized in Table 2. The 11-µl reaction mixture of the phase 1 PCR contained 1 U TrueStart Hot Start *Taq* DNA polymerase, 2.5 x TrueStart *Taq* buffer (both Fermentas, St. Leon-Rot, Germany), 1.9 mM MgCl_2_, 0.25 mM of each dNTP, and 15-121 ng of genomic DNA. Amplification was carried out on a Gene Amp PCR System 9700 (Applied Biosystems, Foster City, CA, USA) according to following protocol: initial denaturation step at 98°C for 1 min; ten cycles of 95 °C/20 sec, 66 °C/2min, 64 °C/2 min, 62 °C/2 min and 72 °C/20 sec; ten cycles of 95 °C/20 sec, 64 °C/2 min, 62 °C/2 min, 60 °C/2 min and 72 °C/20 sec; finally ten cycles of 95 °C/20 sec, 62 °C/2min, 60 °C/2 min, 58 °C/2 min and 72 °C/20 sec.

For the universal primer amplification (step-2 PCR), the products of the step-1 PCR were further amplified using the two universal primers. The components of the reaction mixture were selected according to the recently published guidelines.^41^ The step-2 PCR mixture contained 1x Advantage 2 Polymerase Mix, 1x Advantage 2 SA buffer (both Clontech Laboratories, Mountain View, CA, USA), 0.3 mM of each dNTP, 20 µM of universal primer 1 and 2 and a 4 µl aliquot of the step-1 PCR product and had a final volume of 23 µl. The cycling conditions of the step-2 PCR were: initial denaturation at 98 °C/1 min; 30 cycles of 98 °C/10 sec, 54 °C for 30 sec and 70 °C/60 sec and a final extension step at 70 °C/5 min.

**Ion-pair reversed-phase liquid chromatography-electrospray ionization time-of-flight mass spectrometry (ICEMS)**

After the step-2 PCR the reaction mixture was directly analyzed by ICEMS. An Ultimate fully integrated capillary HPLC system (LC Packings, Amsterdam, The Netherlands) was used for chromatographic separation. Sample injection was accomplished with a Famos microautosampler (LC Packings) equipped with a 2 µl loop. The 45 x 0.2 mm i.d. monolithic capillary column was prepared according to the published protocol.^42^ The flow rate was set to 2.0 µl/min. The column temperature was set to 70 °C to denature the amplicons into the corresponding single-strands. After injection the column was washed with a 25 mM aqueous solution of CycHDMAA containing 5% acetonitrile for 3 min. Separation of the single-stranded DNA molecules was accomplished with a gradient of 20-35% acetonitrile in 25 mM CycHDMAA within 12 min. The eluting nucleic acids were detected online by ESI-MS, which was performed on a QSTAR XL mass spectrometer (AB Sciex, Foster City, CA) equipped with a modified TurboIonSpray source.^30,43^ Mass calibration and optimization of instrumental parameters were performed in the negative ion mode as described previously.^30,43^ The spray voltage was set to -4 kV. Gas flows of 15 arbitrary units (nebulizer gas) and 30 arbitrary units (turbo gas) were employed. The temperature of the turbo gas was adjusted to 200 °C. The accumulation time was set to 1 s. Mass spectra were recorded in the *m/z* range between 800 and 1200 on a personal computer operating with the Analyst QS software (version 1.0, service pack 8 and Bioanalyst extension, AB Sciex).

**Confirmatory sequencing**

To evaluate the reliability of the multiplex genotyping assay, confirmative Sanger sequencing experiments were performed. A detailed description of the sequencing protocol is provided in the Electronic Supplementary Material (Material and Methods S1).

**Results and discussion**

ICEMS represents a reliable tool for the characterization of single- and multiplexed PCRs.^26,27,29^ Genotyping is based on the identification of allele-specific molecular masses within a pool of measured amplicon-specific masses. Thus, a key prerequisite for unequivocal mass spectrometric genotyping is the ability to differentiate all possible amplicon- as well as allele-specific masses. Differentiation of alleles of a certain marker, usually representing a single base exchange within an amplified sequence, can be assured by employing high-resolution molecular mass measurements.^30^ For the typing of multiple SNPs, specificity can be obtained by using staggered amplicon lengths. In the majority of cases, molecular mass interferences can be avoided by using length intervals of 1 or 2 bp between amplicons. In the commonly applied strategy for designing PCR multiplexes, a very narrow T_m_-range is defined for the selection of possible primer sequences. Thus, usually only a limited number of appropriate primer positions next to a targeted SNP come into consideration, which particularly complicates the creation of large PCR multiplexes. Typically, the design of any new assays is a time-consuming and laborious process. Furthermore, the addition of new targets to an existing multiplex can become challenging. Thus, practical solutions in this regard are still desirable. In the present work, we demonstrate how ICEMS genotyping assay design and optimization was markedly facilitated using a novel design and amplification strategy.

**APEX-2 PCR strategy combined with ICEMS**

In search for a simpler and more flexible amplification strategy, the APEX-2 multiplex design and amplification strategy was tested, which employs a “one nucleotide” amplification approach using a two-step PCR protocol including a single (universal) primer PCR.^35^ The compatibility of the APEX-2 protocol with ICEMS genotyping was evaluated by analyzing several multiplex PCRs (Electronic Supplementary Material Table S1). ICEMS analysis of 5-, 10-, 15-, 20-, 25- and 30-plex reactions (amplicon length 79-126 bp) resulted in average genotype call rates of 100%, 95%, 86%, 90%, 74% and 70%, respectively. Hence, the genotype call rate decreased with increasing multiplexing level (Figure 2a). The APEX-2 amplification strategy produced imbalanced multiplexes giving rise to incomplete ICEMS genotyping. These imbalances in PCR efficiency did obviously not affect APEX-2 genotyping, which employs a further reaction step (SBE) before detection takes place. However, in combination with ICEMS genotyping, where the raw PCR products are directly analyzed, imbalances in amplicon concentrations seem to be more critical. A parameter, which was considered to have a particular impact on the detectability of amplicons within a multiplexed PCR, was length of primer and amplicon, respectively. As demonstrated for the 30-plex reaction (Figure 2b), the error rate increased with increasing primer/amplicon length. Typically, undetected amplicons were larger than 100 bp, and their amplification involved specific primers with lengths beyond 30 nts.

ICEMS genotyping using the APEX-2 amplification approach was further complicated by spectral complexity. Parts of the complexity were attributable to the presence of a high level of unspecific mass signals resulting from for instance unspecific primer-primer interactions during amplification (Figure 3a). Furthermore, signals originating from the cleavage of the 3’ terminal bases from the corresponding blunt-ended single strands were found to add to the number of mass signals observed in the deconvoluted mass spectra (Figure 3b).^28,41^

The occurrence of imbalanced amplification and spectral complexity clearly indicates the need for a significant modification of the APEX-2 amplification strategy for ICEMS genotyping.

**Two-step amplification strategy adapted for ICEMS**

Due to the drop-out of the larger sized amplicons and the spectral complexity observed with the original APEX-2 amplification strategy, the design strategy was altered and adapted to the specific requirements of ICEMS genotyping. The newly developed approach comprises three major modifications (Figure 1):

(1) For APEX-2 genotyping, the primers were originally positioned immediately up- and downstream of the polymorphic site of interest. Only the length of the primers varied depending on the genomic context. In the APEX-2 setup primer lengths up to 50 nts were tolerated. Preliminary experiments revealed that amplification involving primers larger than 30 nts lead to imbalanced amplification giving rise to marker drop-out in multiplexed PCRs. Accordingly, the primer length was restricted to 30 nts.

The SNP specific property for ICEMS genotyping is the mass of the corresponding amplicon, which has to be considered when primers and amplicons are designed for a multiplex approach. Thus, to obtain different allele specific masses, the length of the targeted DNA sequence was varied. Instead of solely targeting the SNP of interest, the distance between the primer positions and the SNP of interest was gradually increased. By this means, the required inter-amplicon mass differences were easily obtained without impairing PCR efficiency through extra-elongated primers. Moreover, this strategy enabled a more flexible primer positioning. Thereby an enhanced and more balanced PCR efficiency of the amplicons was achieved.

The developed strategy allows simple and fast design of efficiently working specific primer sequences that can easily be integrated into new or existing PCR multiplexes. In comparison to established design strategies for ICEMS genotyping, flexibility is significantly increased due to the tolerance of a broad range of T_m_-values (55°C-65°C).

(2) For APEX-2 genotyping, a single 21 bp universal sequence for both, the forward and reverse primer, was used. This enabled SBE on microarray of both strands with the same (universal) oligonucleotide. For the ICEMS amplification strategy, two different universal sequences, one to be added to the specific forward primers (US1) and another to be added to the reverse primers (US2), were designed. The aim of this approach was, to avoid hybridization of the forward and reverse strands during PCR. Thereby, the general PCR efficiency was enhanced and undesired PCR side-products were reduced.

(3) The third modification involved the DNA polymerase used. It was anticipated that spectral complexity originating from the cleavage of the 3’ terminal bases from blunt-ended single strands could be reduced by using the Advantage 2 polymerase for amplification.^28,41^

**Development of a 23-plex PCR-ICEMS assay for a clinical pharmacogenetic study**

To assess the compatibility of the newly developed two-step PCR strategy with ICEMS genotyping, a 23-plex assay for a clinical study investigating the pharmacogenetic aspects of opioid replacement therapy was developed. In total, 46 primer were designed, each consisting of a specific and a universal part. The length of the primers varied between 18-28 bp (specific part) and 36-46 bp (specific plus universal part), respectively. The 46 primers were designed without considering the compatibility of the primers (e.g. by using corresponding software tools), T_m_ or GC content. Thereby, the process of primer designing could be remarkably accelerated and facilitated. Mass overlaps of the multiple amplicons were avoided by gradually increasing the length of the amplified DNA target sequence. The problem of inevitable mass overlaps between two amplicons (due to the specific genomic context) could easily be solved by inserting additional nucleotides (non-template addition) between the 5’ end of the specific sequence and the 3’ end of the universal sequence. The size of the 23 amplicons ranged between 78 and 130 bp and the minimum length interval between the designed amplicons was at least 1 bp, in most cases 2 bp. In a first PCR, the 46 primers were combined in one multiplex reaction aiming at a basic amplification of all targeted DNA sequences. The sophisticated PCR cycling protocol included melting temperatures from 58-66°C, thus promoting a basic amplification of the different target DNA sequences despite inhomogeneous annealing temperatures of the corresponding primers. During amplification, also the universal sequence 1 and 2 were incorporated in the sequence of the PCR products. In a next step, a PCR with a universal primer pair was conducted, using the step-1 PCR products as template. This PCR step enabled a uniform amplification of all amplicons under optimized conditions.

The initial analysis of the 23-plex PCRs using uniform concentrations of all primers yielded very satisfying results. Only two of the 46 primers were redesigned due to a relatively poor amplification of the corresponding amplicons (rs7439366, rs2306283). The signal intensities of six more amplicons were slightly below that of the remaining ones. Consequently, we decided to increase the concentrations of the corresponding primer pairs to achieve a more balanced template amplification. After these relatively slight optimization steps, excellent signal to noise ratios were achieved in the deconvoluted mass spectra for all specific mass signals. No further optimization steps (e.g. adjustments of PCR reagent concentrations, cycling conditions or amount of template DNA) were required. Overall, using the modified two-step amplification strategy, the assay design was simple and transparent. Expenditure of time, costs and hands-on work associated with assay development and optimization was significantly lowered compared to previous multiplex PCR-ICEMS assays.

An example for the analysis of an optimized 23-plex PCR is shown in Figure 4. The crude multiplex PCR products were injected onto the chromatographic column. Due to the application of an elevated column temperature (70°C), the double stranded DNA molecules were denatured into the corresponding single strands. The gradient was started with a delay of 3.0 min to enable extended washing of the adsorbed nucleic acids. Separation of the single-stranded DNA molecules was accomplished with a gradient of 20-35% acetonitrile in 25 mM CycHDMAA within 12 min. Single strands corresponding to the targeted amplicons eluted between 12-15.5 min. To facilitate data treatment, the informative part of the peak was divided into 14 sections, each representing a time slot of 15 s. All mass spectra collected within a single time slot were extracted, averaged and deconvoluted to obtain the molecular mass information. The measured molecular masses were used to characterize the nucleotide compositions of the different amplicons. Both DNA single strands served as basis for determining the nucleotide composition of an amplicon, thus increasing the reliability of the mass spectrometric assay. The specific alleles were identified by comparing the measured molecular mass with the theoretical molecular mass. Deviations of the measured mass from the theoretical molecular mass did not exceed the routinely observed measurement error (20–50 ppm).

**Application of the 23-plex PCR-ICEMS assay to pharmacogenetic genotyping**

The developed 23-plex pharmacogenetic genotyping assay was applied to genotype a study population of 284 individuals consisting of opioid dependent individuals and healthy controls. Details of the pharmacogenetic study will be described elsewhere (B. Beer *et al.*, manuscript in preparation). A total of 6469 out of 6532 polymorphic positions (initial genotype call rate of 99.0%) were immediately successfully genotyped. The highest portion of drop-outs (37) was observed for the amplicon rs1042114. All drop-outs were successfully retyped by singleplex PCR. The frequencies of the observed genotypes are summarized in Table 3. The determined allelic frequencies correlated well to values published for other European populations (http://www.ncbi.nlm.nih.gov/snp/). Chi-square testing revealed that the genotype distribution of all except one SNP (*ADRBK2* rs5761122, p = 0.004) were within the Hardy-Weinberg equilibrium.

**Confirmatory sequencing**

Confirmative Sanger sequencing experiments were performed for 6.7% of all ICEMS calls (19 individuals, the 23 candidate positions). Sequencing failed for the SNPs rs6275 and rs1042114, most likely due to the close vicinity of the targeted polymorphisms to the sequencing primers. In the first round of sequencing, 350 of 399 genotypes were determined (88%). Out of these, ten genotypes were not concordant with ICEMS results (Electronic Supplementary Material Table S3). A second round of sequencing was performed, partly with re-designed primers (for the discordant positions, Electronic Supplementary Material Table S2). Finally, ICEMS results were confirmed for all non-concordant positions, except for one case where different homozygous variants were detected (rs11568563). In sum, 99.8% of all genotypes acquired with Sanger sequencing were concordant with genotypes determined by ICEMS.

**Conclusions**

We developed a 23-plex pharmacogenetic genotyping assay by transferring the amplification strategy of a microarray-based genotyping technology to ICEMS genotyping. The novel amplification strategy was associated with facilitated and accelerated assay design and optimization. A clear advantage of the developed two-step amplification strategy over previously used procedures to set up multiplexed PCR assays for ICEMS is that the need for experience in primer design and for empirical refining of the reaction mixture composition as well as thermal cycling conditions is minimized. At the same time, the applied two-step PCR strategy resulted in efficient and quantitatively balanced target amplification – both are very beneficial attributes for the direct analysis of the multiplex PCR products with ICEMS. The 23-plex PCR-ICEMS assay was successfully applied in a pharmacogenetic study including 284 individuals. Thereby, an initial genotype call rate of 99.0% was observed. Overall, the developed genotyping assay turned out to be a very reliable, accurate, convenient and cost-efficient tool particularly useful for pharmacogenetic studies investigating multiple genetic variants.

**Acknowledgments**

This work was supported by the Anniversary Fund of the Oesterreichische Nationalbank (grant number: 13253) and by targeted financing from the Estonian Government (SFO180142s08) and the FP7 grant OPENGENE.

**References**

1. S. Kim, A. Misra, *Annu Rev. Biomed. Eng.*, 2007, **9**, 289-320.

2. C. Ding, S. Jin, *Methods Mol. Biol.*, 2009, **578**, 245-254.

3. B. S. Shastry, *J. Hum. Genet.*, 2005, **50**, 321-328.

4. T. Bernig, S. J. Chanock, *Expert Rev. Mol. Diagn.*, 2006, **6**, 319-331.

5. C. W. Lam, K. C. Lau, S. F. Tong, *Adv. Clin. Chem.*, 2010, **52**, 1-18.

6. C. G. Huber, H. Oberacher, *Mass Spectrom. Rev.*, 2001, **20**, 310-343.

7. H. Oberacher,  *Anal. Bioanal. Chem.*, 2008, **391**, 135-149.

8. H. Oberacher,  *Eur. J. Mass Spectrom.*, 2010, **16**, 351-365.

9. C. Jurinke, D. van den Boom, C. R. Cantor, H. Koster, *Adv. Biochem. Eng. Biotechnol.*, 2002, **77**, 57-74.

10. K. Tang, D. Opalsky, K. Abel, D. van den Boom, P. Yip, G. Del Mistro, A. Braun, C. R. Cantor, *Int. J. Mass Spectrom.*, 2003, **226**, 37-54.

11. M. S. Bray, E. Boewinkle, P. A. Doris, *Hum. Mutat.*, 2001, **17**, 296-304.

12. J. Tost, I. G. Gut, *Mass Spectrom. Rev.*, 2002, **21**, 388-418.

13. J. Tost, I. G. Gut, *J. Mass Spectrom.*, 2006, **41**, 981-95.

14. T. J. Griffin, J. G. Hall, J. R. Prudent, L. M. Smith, *Proc. Natl. Acad. Sci. USA*, 1999, **96**, 6301-6306.

15. J. Mengel-Jorgensen, J. J. Sanchez, C. Borsting, F. Kirpekar, N. Morling, *Anal. Chem.*, 2004, **76**, 6039-6045.

16. S. Kim, M. E. Ulz, T. Nguyen, C. M. Li, T. Sato, B. Tycko, J. Ju, *Genomics* , 2004, **83**, 924-931.

17. S. Sauer, D. H. Gelfand, F. Boussicault, K. Bauer, F. Reichert, I. G. Gut, *Nucleic Acids Res.*, 2002, **30**, 1-5.

18. A. P. Null, J. C. Hannis, D. C. Muddiman, *Anal. Chem.*, 2001, **73**, 4514-4521.

19. R. Sampath, T. A. Hall, C. Massire, F. Li, L. B. Blyn, M. W. Eshoo, S. A. Hofstadler, D. J. Ecker, *Ann. N.Y. Acad. Sci.*, 2007, **1102**, 109-120.

20. C. D. Baldwin, G. B. Howe, R. Sampath, L. B. Blyn, H. Matthews, V. Harpin , T. A. Hall, J. J. Drader, S. A. Hofstadler, M. W. Eshoo, K. Rudnick, K. Studarus, D. Moore, S. Abbott, J. M. Janda, C. A. Whitehouse, *Diagn. Microbiol. Infect. Dis.*, 2009, **63**, 403-408.

21. H. Oberacher, P. J. Oefner, W. Parson, C. G. Huber, *Angew. Chem. Int. Ed.*, 2001, **40**, 3828-3830.

22. B. Berger, G. Hölzl, H. Oberacher, H. Niederstätter, C. G. Huber, W. Parson, *J. Chromatogr. B*, 2002, **782**, 89-97.

23. H. Oberacher, H. Niederstatter, F. Pitterl, W. Parson, *Anal. Chem.*, 2006, **78**, 7816-7827.

24. H. Oberacher, P. J. Oefner, G. Hölzl, A. Premstaller, C. G. Huber, *Nucl. Acids Res.*, 2002, **30**, e67.

25. H. Oberacher, C. G. Huber, P. J. Oefner, *Hum. Mutat.*, 2003, **21**, 86-95.

26. H. Oberacher, F. Pitterl, H. Niederstatter, E. M. Weiss, E. Stadelmann, J. Marksteiner, W. Parson, *Anal. Bioanal. Chem.*, 2006, **386**, 83-91.

27. B. Beer, R. Erb, F. Pitterl, H. Niederstatter, O. Maronas, A. Gesteira, A. Carracedo, I. Piatkov, H. Oberacher, *Anal. Bioanal. Chem.*, 2011, **400**, 2361-2370.

28. F. Pitterl, H. Niederstatter, G. Huber, B. Zimmermann, H. Oberacher, W. Parson, *Electrophoresis*, 2008, **29**, 4739-4750.

29. H. Oberacher, F. Pitterl, G. Huber, H. Niederstatter, M. Steinlechner, W. Parson, *Hum. Mutat.*, 2008, **29**, 427-432.

30. H. Oberacher, H. Niederstätter, B. Casetta, W. Parson, *Anal. Chem.*, 2005, **77**, 4999-5008.

31. A. P. Shuber, V. J. Grondin, K. W. Klinger, *Genome Res.*, 1995, **5**, 488-493.

32. M. C. Edwards, R. A. Gibbs, *PCR Methods Appl.*, 1994, **3**, S65-75.

33. P. Markoulatos, N. Siafakas, M. Moncany, *J. Clin. Lab. Anal.*, 2002, **16**, 47-51.

34. M. Podder, J. Ruan, B. W. Tripp, Z. E. Chu, S. J. Tebbutt, *BMC Med. Genomics*, 2008, **1**, 5.

35. K. Krjutskov, R. Andreson, R. Magi, T. Nikopensius, A. Khrunin, E. Mihailov, V. Tammekivi, H. Sork, M. Remm, A. Metspalu, *Nucleic Acids Res.*, 2008, **36**, e75.

36. K. Krjutskov, T. Viltrop, P. Palta, E. Metspalu, E. Tamm, S. Suvi, K. Sak, A. Merilo, H. Sork, R. Teek, T. Nikopensius, T. Kivisild, A. Metspalu, *Forensic Sci. Int. Genet.*, 2009, **4**, 43-48.

37. L. S. Meuzelaar , O. Lancaster, J. P. Pasche, G. Kopal, A. J. Brookes, *Nat. Methods*, 2007, **4**, 835-837.

38. A. Brandstätter, H. Niederstätter, M. Pavlic, P. Grubwieser, W. Parson, *Forensic Sci. Int.*, 2007, **166**, 164-175.

39. R. Andreson, E. Reppo, L. Kaplinski, M. Remm, *BMC Bioinformatics*, 2006, **7**, 172.

40. R. Andreson, T. Puurand, M. Remm , *Nucleic Acids Res.*, 2006, **34**, W651-655.

41. H. Oberacher, H. Niederstatter, B. Casetta, W. Parson, *J. Am. Soc. Mass Spectrom.*, 2006, **17**, 124-129.

42. A. Premstaller, H. Oberacher, C. G. Huber, *Anal. Chem.*, 2000, **72**, 4386-4393.

43. H. Oberacher, H. Niederstatter, W. Parson, *J. Mass Spectrom.*, 2005, **40**, 932-945.

**Table 1.** Genes and SNPs that were selected to be included in the pharmacogenetic assay.

| Gene | Symbol | SNP ID | Alleles |
| --- | --- | --- | --- |
| Opioid receptor, mu-1 | *OPRM1* | rs1799971 | A/G |
|  |  | rs9479757 | G/A |
|  |  | rs3778151 | C/T |
|  |  | rs510769 | A/G |
| Opioid receptor, delta-1 | *OPRD1* | rs2236861 | C/T |
|  |  | rs1042114 | T/G |
| Opioid receptor, kappa-1 | *OPRK1* | rs1051660 | G/T |
| Catechol-O-methyltransferase | *COMT* | rs4680 | G/A |
| Signal transducer and activator of transcription 6 | *STAT6* | rs841718 | C/T |
| ATP-binding cassette, subfamily B, member 1 | *ABCB1* | rs2032588 | C/T |
|  |  | rs2032582 | G/T |
|  |  | rs1045642 | C/T |
| Dopamine receptor D2 | *DRD2* | rs6275 | C/T |
| Galanin | *GAL* | rs948854 | A/G |
| Solute carrier organic anion transporter family, 1B1 | *SLCO1B1* | rs4149056 | T/C |
|  |  | rs2306283 | C/T |
| Solute carrier organic anion transporter family, 1A2 | *SCLO1A2* | rs11568563 | A/C |
|  |  | rs45502302 | A/T |
| Melanocortin 1 receptor | *MC1R* | rs1805007 | C/G/T |
|  |  | rs1805008 | C/T |
| Uridine diphosphate glycosyltransferase 2B7 | *UGT2B7* | rs7439366 | C/T |
| 5-Alpha hydroxytryptamine receptor 1A | *5-HTR1A* | rs6295 | C/G |
| Beta-adrenergic receptor kinase 2 | *ADRBK2* | rs5761122 | G/A |

**Table 2.** Specific sequences and final concentrations of the primers used in the 23-plex PCR assay. To the 5’ end of the specific sequence of the forward (F) and the reverse primers (R) the universal sequence (US1 or 2) was added.

| SNP | Amplicon length [bp] | Specific part of the primer sequence (5’-3’) of the forward (F) and reverse (R) primers | c [µM] |
| --- | --- | --- | --- |
| rs6275 | 78 | F: US1-GTCGGGAGTGCTGTGGAGAC  R: US2-CTCCCCGACCCGTCCCACCA | 0.7 |
| rs1042114 | 80 | F: US1-ACGCATTGGCGCCAGCGCTG  R: US2-GGACGCCTACCCTAGCGCCT | 0.7 |
| rs1799971 | 82 | F: US1-ACCGCATGGGTCGGACAG  R: US2-GGTCAACTTGTCCCACTTAGATG | 0.7 |
| rs2032588 | 84 | F: US1-GCAACATCAGAAAGATGTGCAATG  R: US2-AGACCCTGCGGTGATCAGCAGT | 0.7 |
| rs4680 | 86 | F: US1-GTCAGGCATGCACACCTTGT  R: US2-AGCGGATGGTGGATTTCG | 0.7 |
| rs1051660 | 90 | F: US1-CTCGGGGCGCAGGTAGGG  R: US2-GGACTCCCCGATCCAGATCTTC | 2.1 |
| rs948854 | 92 | F: US1-CTGCAGAGTCACAGGAACGTG  R: US2-GCAGAAGGACAGCCGAGAG | 0.7 |
| rs4149056 | 98 | F: US1-CCCCTATTCCACGAAGCATATTA  R: US2-GGAATCTGGGTCATACATGTGG | 0.7 |
| rs1805008 | 100 | F: US1-ACGACACTGGCCACCCAGAT  R: US2-ACAGCATCGTGACCCTGCCG | 0.7 |
| rs1045642 | 102 | F: US1-GACTCGATGAAGGCATGTATGT  R: US2-GGTGGTGTCACAGGAAGAGA | 0.7 |
| rs6295 | 104 | F: US1-CAATTATTGCTAATTGATGGAAGAAG  R: US2-CGAGAACGGAGGTAGCTTTTTA | 2.1 |
| rs2032582 | 106 | F: US1-CAATCATATTTAGTTTGACTCACCTTCC  R: US2-CTGGACAAGCACTGAAAGATAAGA | 0.7 |
| rs510769 | 108 | F: US1-TTGATATTGATTGTGTTGGTGTTGA  R: US2-CAGATATATGGCATTTCACATTCACA | 2.1 |
| rs5761122 | 110 | F: US1-CCTGAAAAGGACAAGAGGAGTG  R: US2-TCAGCTTTCTGCTGGCAGACG | 0.7 |
| rs9479757 | 112 | F: US1-TGCTTATGACATCACCAACATATCA  R: US2-ACAGGCAAGGTGAGTGATGTTACC | 0.7 |
| rs45502302 | 114 | F: US1-GAAGTAGACAACCTGATGGATCCTG  R: US2-CCTCAAACAGTTTCTTGTGTATGG | 0.7 |
| rs2306283 | 116 | F: US1-TTCAGTGATGTTCTTACAGTTACAGG  R: US2-GGATAAGGTCGATGTTGAATTTTCTGA | 2.1 |
| rs2236861 | 118 | F: US1-TGACTGTTGTTTCTTGTCAAGCTCT  R: US2-CTGCACAAACACAGGGACGA | 0.7 |
| rs11568563 | 120 | F: US1-TATAGGAAATACCCAAAGGCAGGAT  R: US2-CATTAATGTGGGTGTACGTCCTAGT | 0.7 |
| rs7439366 | 121 | F: US1-CAAAATCAACATTTGGTAAGAGTGGAT  R: US2-ATAATGGGGAAAGCTGACGTATG | 0.7 |
| rs1805007 | 124 | F: US1- GGGTCACGATGCTGTGGTAG  R: US2- CTGTCCAGCCTCTGCTTCCT | 2.1 |
| rs3778151 | 126 | F: US1-TTTCTATTGTGTTCTGGAGCTTGG  R: US2-GTGAATGCAATCTTTCATTTTCAAG | 2.1 |
| rs841718 | 130 | F: US1-AAGTAAGAGAAGCACAGCTATGA  R: US2-ACCCACTTCCTCCCTGCTC | 0.7 |

**Table 3.** Observed genotype frequencies in a study population of 284 individuals.

| SNP ID | Gene | Genotype frequency | SNP ID | Gene | Genotype frequency |
| --- | --- | --- | --- | --- | --- |
| rs1799971 | *OPRM1* |  | rs7439366 | *UGT2B7* |  |
| A/A |  | 0.75 | C/C |  | 0.20 |
| A/G |  | 0.24 | C/T |  | 0.52 |
| G/G |  | 0.01 | T/T |  | 0.28 |
| rs9479757 | *OPRM1* |  | rs2032588 | *ABCB1* |  |
| G/G |  | 0.88 | C/C |  | 0.89 |
| G/A |  | 0.12 | C/T |  | 0.11 |
| A/A |  | 0 | T/T |  | 0 |
| rs3778151 | *OPRM1* |  | rs1045642 | *ABCB1* |  |
| C/C |  | 0.03 | C/C |  | 0.22 |
| C/T |  | 0.21 | C/T |  | 0.56 |
| T/T |  | 0.77 | T/T |  | 0.22 |
| rs510769 | *OPRM1* |  | rs4149056 | *SLCO1B1* |  |
| A/A |  | 0.06 | T/T |  | 0.74 |
| A/G |  | 0.29 | T/C |  | 0.23 |
| G/G |  | 0.66 | C/C |  | 0.03 |
| rs2236861 | *OPRD1* |  | rs2306283 | *SLCO1B1* |  |
| C/C |  | 0.60 | C/C |  | 0.16 |
| C/T |  | 0.34 | C/T |  | 0.44 |
| T/T |  | 0.06 | T/T |  | 0.40 |
| rs1042114 | *OPRD1* |  | rs11568563 | *SCLO1A2* |  |
| T/T |  | 0.76 | A/A |  | 0.90 |
| T/G |  | 0.24 | A/C |  | 0.10 |
| G/G |  | 0 | C/C |  | 0 |
| rs1051660 | *OPRK1* |  | rs45502302 | *SCLO1A2* |  |
| G/G |  | 0.79 | A/A |  | 1.00 |
| G/T |  | 0.20 | A/T |  | 0 |
| T/T |  | 0.01 | T/T |  | 0 |
| rs5761122 | *ADRBK2* |  | rs948854 | *GAL* |  |
| G/G |  | 0.49 | A/A |  | 0.59 |
| G/A  A/A |  | 0.47 | A/G |  | 0.31 |
| A/A |  | 0.04 | G/G |  | 0.10 |
| rs841718 | *STAT6* |  | rs6295 | *5-HTR1A* |  |
| C/C |  | 0.21 | C/C |  | 0.26 |
| C/T |  | 0.50 | C/G |  | 0.50 |
| T/T |  | 0.29 | G/G |  | 0.24 |
| rs4680 | *COMT* |  | rs6275 | *DRD2* |  |
| G/G |  | 0.20 | C/C |  | 0.48 |
| G/A |  | 0.54 | C/T |  | 0.40 |
| A/A |  | 0.26 | T/T |  | 0.12 |
| rs1805007 | *MC1R* |  | rs2032582 | *ABCB1* |  |
| C/C |  | 0.90 | G/G |  | 0.34 |
| C/T |  | 0.10 | G/T |  | 0.48 |
| T/T | 0000000transcription 6 | 0 | T/T |  | 0.16 |
| rs1805008 | *MC1R* |  | G/A |  | 0.01 |
| C/C |  | 0.88 | T/A |  | 0.02 |
| C/T |  | 0.11 | A/A |  | 0 |
| T/T |  | 0.01 |  |  |  |

**Figures**

**Figure 1.** Comparison of (a) the APEX-2 and (b) the ICEMS-specific primer design strategy.

**Figure 2.** Influence of (a) the multiplexing level and (b) the amplicon length within a 30-plex PCR on the genotyping efficiency of ICEMS. Multiplexed PCR were designed with the APEX-2 strategy.

**Figure 3.** (a) Unspecific mass signals and (b) signals originating from the cleavage of the 3’ terminal bases from blunt-ended single strands were observed in deconvoluted mass spectra obtained from multiplexed PCR designed with the APEX-2 strategy.

**Figure 4.** Analysis of the 23 simultaneously amplified PCR amplicons with ICEMS. (a) Reconstructed ion chromatogram, (b) zoom on the peak resulting from the elution of the denatured PCR products, (c) mass spectra obtained by deconvolution of the raw mass spectra extracted from 14 segments each representing a time slot of 15 s. The obtained molecular masses were used to assign the allelic states of the genotyped SNPs. .


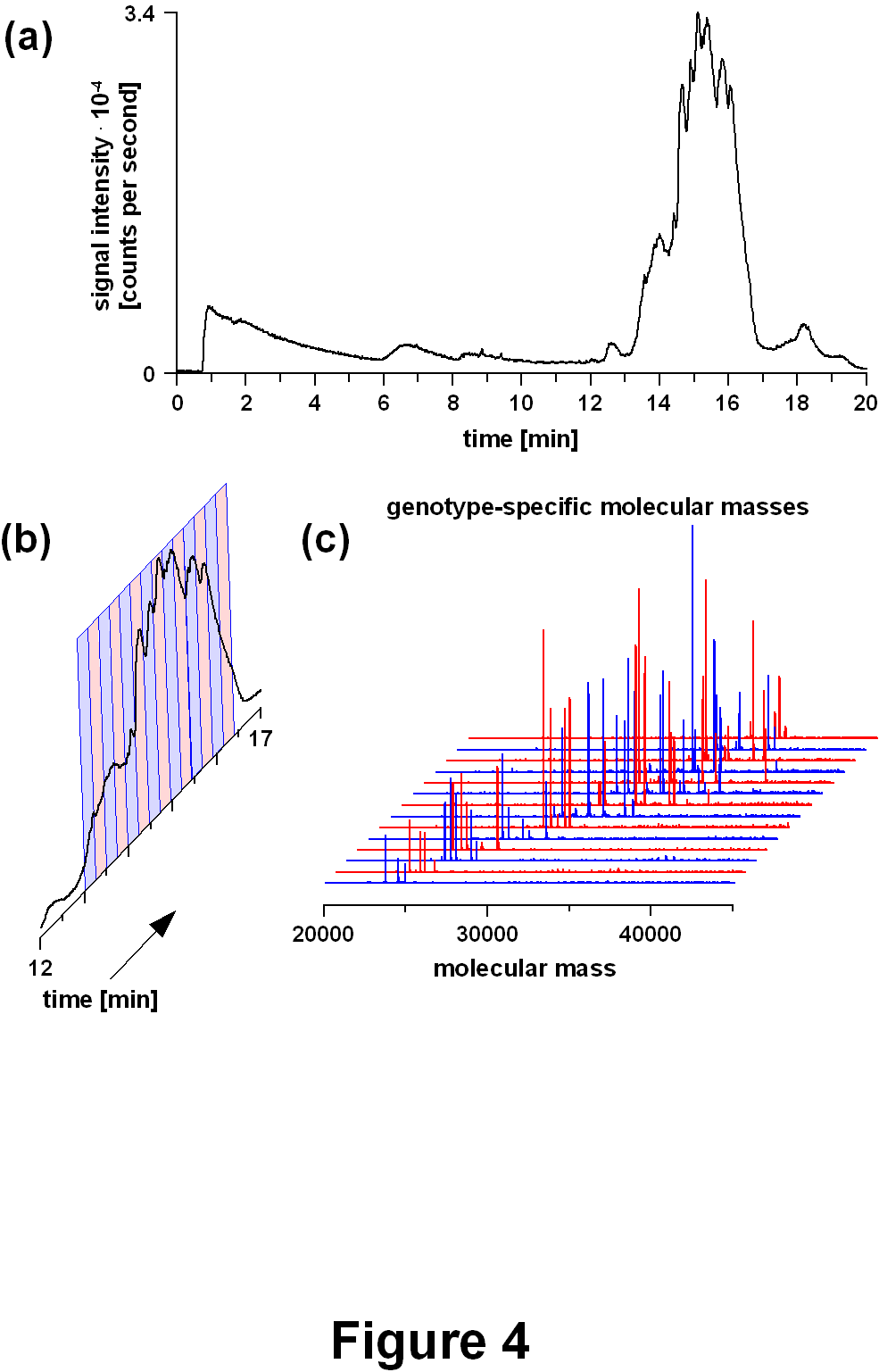


**Electronic Supplementary Material**

**Table S1.** Different APEX-2 multiplex sets generated for ICEMS analysis.

| **CALL RATE (%)** | |  |  |  |  |
| --- | --- | --- | --- | --- | --- |
| 100 | 95 | 86 | 90 | 74 | 70 |
|  |  |  |  |  |  |
| **AMPLICON LENGTH** | | |  |  |  |
| **5-plex** | **10-plex** | **15-plex** | **20-plex** | **25-plex** | **30-plex** |
| 79 | 79 | 79 | 79 | 79 | 79 |
| 89 | 89 | 89 | 89 | 89 | 89 |
| 100 | 100 | 100 | 100 | 100 | 100 |
| 110 | 110 | 110 | 110 | 110 | 110 |
| 126 | 126 | 126 | 126 | 126 | 126 |
|  | 84 | 84 | 84 | 84 | 84 |
|  | 94 | 94 | 94 | 94 | 94 |
|  | 104 | 104 | 104 | 104 | 104 |
|  | 107 | 107 | 107 | 107 | 107 |
|  | 115 | 115 | 115 | 115 | 115 |
|  |  | 82 | 82 | 82 | 82 |
|  |  | 92 | 92 | 92 | 92 |
|  |  | 97 | 97 | 97 | 97 |
|  |  | 112 | 112 | 112 | 112 |
|  |  | 121 | 121 | 121 | 121 |
|  |  |  | 86 | 86 | 86 |
|  |  |  | 88 | 88 | 88 |
|  |  |  | 95 | 95 | 95 |
|  |  |  | 102 | 102 | 102 |
|  |  |  | 122 | 122 | 122 |
|  |  |  |  | 80 | 80 |
|  |  |  |  | 99 | 99 |
|  |  |  |  | 108 | 108 |
|  |  |  |  | 113 | 113 |
|  |  |  |  | 119 | 119 |
|  |  |  |  |  | 83 |
|  |  |  |  |  | 87 |
|  |  |  |  |  | 98 |
|  |  |  |  |  | 103 |
|  |  |  |  |  | 123 |

**Material and Methods S1. Confirmatory sequencing experiments.**

The 23 candidate regions were sequenced in 19 individuals (6.7 % of all study subjects). The studied regions were amplified by PCR in a 15 µl volume containing 1× Reaction Buffer B (Naxo, Tartu, Estonia), 0.25 mM of each dNTP (Fermentas), 2.5 mM MgCl_2_, 1.25 U Hot Fire Polymerase (Solis Biodyne, Tartu, Estonia), 1.2 µM of each primer (Metabion) and 33–56 ng of template DNA. For this purpose, the same primers were used as for the multiplex PCR protocol. After the first round of both strand sequencing, six primer pairs were re-designed (Table S2). Cycling was performed with a GeneAmp PCR System 2700 thermocycler (Life Technologies) under the following conditions: 95˚C/15 min (initial denaturation), 10 cycles of 95˚C/30 sec, 64˚C/30 sec, 62˚C/30 sec, 60˚C/30 sec and 72˚C/20 sec, 27 cycles of 95˚C/30 sec, 55˚C/30 sec and 72˚C/20 sec 64˚C, and 72˚C/1 min.

PCR product purification was carried out with Exonuclease I and Shrimp Alkaline Phosphatase treatment. Ten units of ExoI and 1 U SAP (both Fermentas) were added to 5 µl of PCR product and incubated at 37˚C/20 min and at 80˚C/15 min. The BigDye Terminator v3.1 Cycle Sequencing Kit (Life Technologies) was applied in a 10 µl reaction volume containing 1 µl of purified PCR product, 0.7 µl BigDyeTerminator Ready Reaction Premix, 2 µl of BigDye Terminator Sequencing Buffer and 0.9 µM sequencing primer (universal primer 1 and 2). Cycling was carried out by a GeneAmp PCR System 2700 thermocycler under following conditions: 95˚C/20 sec, 30 cycles of 50˚C/15 sec, and 60˚C/1 min. The extension products were purified according to dextran/ethanol precipitation protocol. The purified pellet was dissolved in 10 µl of formamide, followed by electrophoresis on the ABI Prism 3730 DNA Analyzer (Life Technologies).

**Table S2.** Sequences of the newly designed sequencing primers.

| SNP | Fw | Rev |
| --- | --- | --- |
| rs4680 | GATCCAAGTTCCCCTCTCT | GGGCCTGGTGATAGTGG |
| rs2306283 | CTGTAAGAGTCAAATGTTTTTCC | GGGAAATTGACAGAAAGTACTCT |
| rs1805008 | CACTCACCCATGTACTGCTTCA | AGGATGGTGAGGGTGACAGC |
| rs9479757 | TTGATCGATACATTGCAGTC | ATTTGCCATGTAGTCAGCC |
| rs11568563 | ACAAGGGGCCATGGTCATA | TTTTATGGAAGGCCAACTGTG |
| rs7439366 | TTGCCTACATTTTTGCCTAC | TCCCATCTTTCTTTCAGTGT |

**Table S3.** Comparison of the genotypes determined by ICEMS and those assessed in the first and second round of Sanger sequencing.
